# Extracellular arginine availability modulates eIF2α O-GlcNAcylation and heme oxygenase 1 translation for cellular homeostasis

**DOI:** 10.1186/s12929-023-00924-4

**Published:** 2023-05-22

**Authors:** Yu-Wen Hung, Ching Ouyang, Xiaoli Ping, Yue Qi, Yi-Chang Wang, Hsing-Jien Kung, David K. Ann

**Affiliations:** 1 Department of Diabetes Complications and Metabolism, Arthur Riggs Diabetes & Metabolism Research Institute, Beckman Research Institute of City of Hope, City of Hope Comprehensive Cancer Center, Duarte, CA 91010-3000 USA; 2 Department of Computational and Quantitative Medicine, Beckman Research Institute of City of Hope, Duarte, CA 91010 USA; 3 Cancer Center, School of Medicine, University of California, Davis, CA 95817 USA; 4 Irell & Manella Graduate School of Biological Sciences, Beckman Research Institute of City of Hope, Duarte, CA 91010 USA

**Keywords:** Arginine, O-GlcNAcylation, Eukaryotic initiation factor 2α, Protein translation, Heme oxygenase 1, Antioxidant defense

## Abstract

**Background:**

Nutrient limitations often lead to metabolic stress during cancer initiation and progression. To combat this stress, the enzyme heme oxygenase 1 (HMOX1, commonly known as HO-1) is thought to play a key role as an antioxidant. However, there is a discrepancy between the level of HO-1 mRNA and its protein, particularly in cells under stress. O-linked β-N-acetylglucosamine (O-GlcNAc) modification of proteins (O-GlcNAcylation) is a recently discovered cellular signaling mechanism that rivals phosphorylation in many proteins, including eukaryote translation initiation factors (eIFs). The mechanism by which eIF2α O-GlcNAcylation regulates translation of HO-1 during extracellular arginine shortage (ArgS) remains unclear.

**Methods:**

We used mass spectrometry to study the relationship between O-GlcNAcylation and Arg availability in breast cancer BT-549 cells. We validated eIF2α O-GlcNAcylation through site-specific mutagenesis and azido sugar N-azidoacetylglucosamine-tetraacylated labeling. We then evaluated the effect of eIF2α O-GlcNAcylation on cell recovery, migration, accumulation of reactive oxygen species (ROS), and metabolic labeling during protein synthesis under different Arg conditions.

**Results:**

Our research identified eIF2α, eIF2β, and eIF2γ, as key O-GlcNAcylation targets in the absence of Arg. We found that O-GlcNAcylation of eIF2α plays a crucial role in regulating antioxidant defense by suppressing the translation of the enzyme HO-1 during Arg limitation. Our study showed that O-GlcNAcylation of eIF2α at specific sites suppresses HO-1 translation despite high levels of *HMOX1* transcription. We also found that eliminating eIF2α O-GlcNAcylation through site-specific mutagenesis improves cell recovery, migration, and reduces ROS accumulation by restoring HO-1 translation. However, the level of the metabolic stress effector ATF4 is not affected by eIF2α O-GlcNAcylation under these conditions.

**Conclusions:**

Overall, this study provides new insights into how ArgS fine-tunes the control of translation initiation and antioxidant defense through eIF2α O-GlcNAcylation, which has potential biological and clinical implications.

**Supplementary Information:**

The online version contains supplementary material available at 10.1186/s12929-023-00924-4.

## Background

Dysregulated protein synthesis is a hallmark of cancer [1–3], with key roles in aberrant cell proliferation, survival, angiogenesis, metabolism, immune responses, and chemoresistance. For example, oncogenic signaling (e.g., PI3K/AKT/mTOR; Ras/ERK) leads to hyperactive cap-dependent translation, an exquisitely regulated process that directs ribosomes to the initiation codon in the mRNA template [4, 5]. Results from several studies have shown that tumor phenotype is driven not only by global changes in protein synthesis, but also by selective changes in translational efficiency [6]. In addition, tumor cells are capable of reprogramming the proteome to sustain the transformed phenotype and promote their survival in response to nutrient availability and environmental stress [7, 8]. Such selective changes in translation can be achieved by reprogramming the activity of translation initiation in response to specific stress signals.

The eukaryotic initiation factor (eIF) 2 is at the nexus controlling translation initiation efficiency [9]. eIF2α (eIF2α, encoded by *EIF2S1* gene) forms the ternary complex, comprised of eIF2α, β, and γ, methionine (Met)-tRNAi, and GTP. The ternary complex brings the initiator Met-tRNAi to the P site of the 40S ribosomal subunit, initiating protein synthesis. There are two distinct configurations of eIF2, the GDP- or GTP-bound forms. Following the recognition of the start codon by the 43S preinitiation complex, the GTP bound to eIF2 is hydrolyzed to deliver Met-tRNAi and the eIF2-GDP is subsequently released from the 40S subunit and recycled to allow delivery of another Met-tRNAi [10]. In response to metabolic stress, overall protein synthesis is reduced to relieve cells from endoplasmic reticulum (ER) stress [11]. Notably, the phosphorylation of eIF2α at serine (Ser) 51 effectively reduces the level of active eIF2, significantly inhibiting mRNA translation initiation [12] and global protein synthesis [1] while promoting selective translation of proteins in response to the stresses, such as activating transcription factor 4 (ATF4), representing a major mechanism in ER stress response [13]. Among the various targets transcriptionally activated by ATF4, heme oxygenase 1 (HO-1, encoded by *HMOX1* gene) is one of the most extensively studied stress-induced antioxidant enzymes [14]. Increased levels of HO-1 ameliorate ER stress-associated oxidative stress [15] and dampens oxidative stress to promote cell escaping from radiation- or chemo-therapies [16]. Genetic or pharmacological upregulation of HO-1 significantly promotes the survival and metastasis of many cancers including breast cancer, melanoma, chronic myelogenous leukemia, and others [17–20]. While the role of the HO-1 antioxidant stress system in the ER stress response is well-established, its control of HO-1 protein translation has yet to be fully understood.

Protein O-linked N-acetylglucosaminylation (O-GlcNAcylation), a dynamic, inducible posttranslational modification (PTM) of intracellular proteins, is controlled by the concerted actions of a single pair of opposing enzymes, termed O-linked N-acetylglucosamine (O-GlcNAc) transferase (OGT) and O-GlcNAcase (OGA) [21, 22]. Together, these two enzymes cycle O-GlcNAc provided by the hexosamine biosynthetic pathway (HBP), a major pathway of glucose (Glc) metabolism [21, 22]. OGT transfers a single O-GlcNAc moiety to the hydroxyl group of Ser or threonine (Thr) residues of substrate proteins from the direct donor uridine diphosphate N-acetylglucosamine (UDP-GlcNAc), whereas OGA catalyzes the opposite reaction to cleave O-GlcNAc from O-GlcNAcylated proteins [21, 22]. Protein O-GlcNAcylation is increasingly recognized as an important cellular regulatory mechanism in all aspects of cellular function, including metabolism, signal transduction, transcriptional regulation, DNA repair, cell cycle control, protein trafficking, and regulation of the cell structure [21, 23–31]. Notably, O-GlcNAcylation, like phosphorylation and other PTMs, alters the tertiary structure, blocks ligand interactions, competes with other PTMs, and/or controls enzyme activity [21]. Consequently, deregulation of O-GlcNAc cycling has been implicated in the pathogenesis of a plethora of chronic diseases, including human cancer [30]. Identification of O-GlcNAc-modified substrates will not only lead to a better understanding of the functions of protein O-GlcNAcylation but will also be helpful in the development of new strategies for treating and preventing human diseases. However, the impact of O-GlcNAcylation on translation initiation during ER stress response to different metabolic stresses, especially arginine (Arg) shortage (ArgS), remains to be understood.

Arg is one of the most versatile amino acids that participates in multiple metabolic pathways in highly proliferative cells [32]. Our laboratory has a longstanding interest in the effects of Arg deprivation therapy on breast and prostate cancers, as demonstrated in our previous studies [7, 33–36]. Our research showed that ArgS leads to a decrease in glycolysis and a shift in metabolic pathways. Although only a small portion of Glc enters the HBP pathway [30], O-GlcNAcylation is a rapidly growing cellular signaling mechanism that rivals protein phosphorylation in many proteins [30]. HBP plays a central role in sensing the availability of intracellular Glc, glutamine (Gln), acetyl-CoA and UTP in cancer cells via producing UDP-GlcNAc, a substrate for protein O-GlcNAcylation (Additional file 1: Fig. S1, [37]). A crucial unanswered question is whether the limited availability of Arg, a non-component of HBP, has an impact on protein O-GlcNAcylation.

In this study, we used the unbiased screen of O-GlcNAc-modified proteins to examine the cellular response to ArgS. In this study, we describe eIF2α as a key target of ArgS-induced O-GlcNAcylation. We show that eIF2α O-GlcNAcylation suppresses HO-1 protein synthesis, independent of eIF2α Ser51 phosphorylation and ATF4 induction. Our findings uncouple the conventional link between eIF2α Ser51 phosphorylation and protein translation inhibition, a hallmark of canonical integrated stress response (ISR) to ER Stress, during ArgS. Overall, we define eIF2α O-GlcNAcylation as a new mechanism of stress response in cells experiencing ArgS. Importantly, eIF2α O-GlcNAcylation alone is sufficient to suppress the synthesis of HO-1, regardless of eIF2α Ser51 phosphorylation. Based on our findings that elevation of HO-1 expression leads to a better recovery from ArgS, we propose that suppression of HO-1 translation by ArgS-mediated mechanisms may provide therapeutic benefits against cancer [32].

## Materials and methods

### Cell line, cell culture, and treatment

Human triple-negative breast cancer BT-549, MDA-MB-231, Hs578T, MDA-MB-468, and MDA-MB-435, human estrogen receptor- and progesterone receptor-positive breast cancer MCF7, and human embryonic kidney (HEK) 293T (HEK293 cells with SV40 T-antigen) cells were originally purchased from the American Type Culture Collection (ATCC, Manassas, VA, USA). Cells were cultured in Dulbecco’s Modified Eagle’s Medium (DMEM; Thermo Fisher Scientific, Waltham, MA, USA) with 4.5 g/L Glc supplemented with 10% (vol/vol) fetal bovine serum (FBS; Thermo Fisher Scientific), 1% (vol/vol) Penicillin–Streptomycin (10,000 units/ml of penicillin, and 10,000 μg/ml of streptomycin; Thermo Fisher Scientific). All cells were maintained at 37 °C in a 5% (vol/vol) CO_2_, 95% (vol/vol) air incubator.

In this study, various treatments were used to investigate the role of specific pathways in cellular processes. Proteasome activity was inhibited by treating cells with MG132 ([38], 10 μM; Tocris Bioscience, Minneapolis, MN, USA) for 24 h. HO-1 activity was activated with cobalt protoporphyrin (CoPPIX) ([39], 12.5 μM; MilliporeSigma, Burlington, MA, USA) and inhibited with Zinc protoporphyrin-9 (ZnPPIX) ([40], 5 μM; MilliporeSigma), respectively, both for 24 h. ER stress was induced by treating cells with tunicamycin (TN) ([41], 2 μg/ml; MilliporeSigma) for 24 h, while TUDCA ([42], 200 or 400 μM; FOCUS biomolecules, Plymouth Meeting, PA, USA) was used to inhibit ER stress for the same duration. Additionally, to disrupt the interaction between p-eIF2α and eIF2B, an ISR inhibitor (ISRIB) ([43], 200 or 400 nM; MilliporeSigma) was used to treat cells for 24 h. These treatments were applied to investigate specific molecular pathways and their effects on cellular processes.

To culture cells in various nutrient withdrawal conditions, we used Arg-free (-Arg) DMEM (Thermo Fisher Scientificsupplemented with 10% (vol/vol) dialyzed FBS (Gemini Bio; Sacramento, CA, USA), 1% (vol/vol) Penicillin–Streptomycin and 146 mg/L L-lysine (MilliporeSigma) to achieve Arg withdrawal. Cells were initially cultured in the complete medium until desired cell density was reached. The complete medium was aspirated off and the cells were washed briefly with phosphate-buffered saline (PBS) and continued to be incubated in the -Arg medium for 24 or 48 h, based on the experimental design at 37 °C prior to harvesting. For the ArgS treatment, cells were seeded in 12-well plates and treated with Arg deiminase pegylated with 20,000-molecular-weight polyethylene glycol (ADI-PEG20) ([44], 1 μg/ml; Polaris Pharmaceuticals, San Diego, CA, USA) in the complete medium for 24, 48, and 72 h, depending on the experimental design, as described previously [7, 33]. To achieve Glc withdrawal, Glc-free medium was prepared from the DMEM (without Glc and sodium pyruvate; Thermo Fisher Scientific) supplemented with 10% (vol/vol) dialyzed FBS, 1% (vol/vol) Penicillin–Streptomycin, and 1 mM sodium pyruvate (Thermo Fisher Scientific). Similarly, Gln-free medium was prepared from the DMEM (without Glc, Gln, and sodium pyruvate; Thermo Fisher Scientific) supplemented with 10% (vol/vol) dialyzed FBS, 1% (vol/vol) Penicillin–Streptomycin, 1 mM sodium pyruvate, and 25 mM Glc (MilliporeSigma). Cells were washed twice with PBS, followed by continued incubation in Glc- or Gln-free medium for 24 or 48 h, depending on experimental design.

### Immunoblotting

Cells were lysed in Laemmli sample buffer (0.045 M Tris–HCl pH 6.8, 10% glycerol, 1% sodium dodecyl-sulfate (SDS), 0.01% bromophenol blue, 0.05 M dithiothreitol (DTT) supplemented with PhosSTOP™ phosphatase inhibitor cocktail (1X; Roche, Basel, Switzerland) and OGA inhibitor-Thiamet G (1 μM; MilliporeSigma) prior to use. The cell lysates in the sample buffer were then boiled for 10–15 min. Protein concentrations were determined by the Bradford assay reagent (BioRAD). Samples were separated by SDS polyacrylamide (12 or 12.5% of acrylamide) gel electrophoresis and separated protein species were subsequently transferred to a polyvinylidene fluoride transfer membrane. Each membrane was horizontally split into multiple strips, based on prestained protein ladder with a broad molecular weight (10–245 kDa), to simultaneously probe for different proteins with desired antibodies. The membranes were then blocked with 5% nonfat dry milk and 0.05% Tween 20 in PBS) for 1 h at room temperature and then probed with specific primary antibodies in 5% BSA and 0.05% Tween 20 in PBS at 4 °C overnight. Blots were washed with PBST (0.05% Tween 20 in PBS) three times (10 min each) and then incubated with the appropriate secondary antibodies for 1 h at room temperature prior to visualization using Versadoc 3000 Imaging System (BioRAD) and quantified by Image Lab software (BioRAD). All the antibodies used in present study were listed in Additional file 2: Table S2.

### Metabolic labeling with N-azidoacetylglucosamine tetraacylated (GlcNAz) and biotin enrichment

The O-GlcNAz labeling of proteins was performed using the azido sugar GlcNAz (Thermo Fisher Scientific) dissolved in DMSO to create a stock solution (10 mM). Cells were incubated in medium containing GlcNAz (50 μM) for 48 h. Cell lysis buffer (Cell Signaling) was used for sample collection and was supplemented with a PhosSTOP™ phosphatase inhibitor cocktail (1X) and an OGA inhibitor (1 μM) prior to use. Freshly prepared cell lysis buffer (1 ml) was added to each 10-cm culture dish, and the dishes were then placed on ice for 5 min. The cell lysates were then transferred into Eppendorf tubes and rotated at 4 °C for an additional 15–20 min to maximize lysis. The lysates were collected by centrifugation at 14,000 × g at 4 °C for 10 min and the supernatant containing the proteins was collected. The protein concentration of the collected supernatant was determined using Bradford assay reagent. To pull down O-GlcNAz-modified proteins, 1.5–2 mg equivalent of whole cell extracts in an adjusted volume to 300 μl of cell lysis buffer were used. To biotinylate O-GlcNAz-modified proteins, phosphine-polyethylene glycol (PEG)3-Biotin (Thermo Fisher Scientific) was added to the protein lysates to a final concentration of 200 μM. The samples were inverted several times and incubated at 37 °C for 2 h. Unreacted phosphine-PEG3-Biotin reagent was removed by chloroform/methanol precipitation. 600 μl of methanol and 300 μl of chloroform were added to each sample, and the samples were vortexed briefly. 300 μl of ddH_2_O was added, and the samples were vortexed again. The samples were then centrifuged at 15,000xg for 5 min, and the aqueous phase was discarded. The resulting protein pellet was washed with methanol (1 ml) twice and resuspended in lysis buffer (200 μl) containing PhosSTOP™ phosphatase inhibitor cocktail (1X) and OGA inhibitor (1 μM). O-GlcNAz-modified biotinylated proteins were pulled down by High-Capacity Streptavidin Agarose (Thermo Fisher Scientific) (30 μl) by incubating overnight with end-over-end rotation at 4 °C. The agarose beads were then washed with PBS buffer five times to remove any non-specifically bound proteins. The GlcNAz-modified proteins were eluted by adding sample buffer (0.045 M Tris–HCl pH 6.8, 10% glycerol, 1% SDS, 0.01% bromophenol blue, 0.05 M DTT, 1X PhosSTOP™ phosphatase inhibitor cocktail, and 1 μM OGA inhibitor to the beads and incubating for 15 min at 100 °C. The eluted O-GlcNAz-modified proteins were then analyzed by immunoblotting using appropriate antibodies against the proteins of interest.

### Sample preparation and proteomic analysis of O-GlcNAz-labeled proteins

The O-GlcNAz-labeled proteins were prepared and enriched by pull-down with magnetic beads. The beads were washed with PBS buffer five times and the supernatant was discarded. The O-GlcNAz proteomics analysis and identification were conducted by the Harvard Center for Mass Spectrometry at Harvard University. Samples were digested on the beads with tetraethylammonium bromide (TEAB) and trypsin. Briefly, 500 μl of 50 mM TEAB buffer was added and heated at 95 °C for 5 min, then cooled to room temperature before being digested with trypsin for 3 h. Samples were analyzed on an Orbitrap Lumos mass spectrometer from Thermo Fisher Scientific, equipped with a dual pump Ultimate 3000 nanoLC system also from Thermo Fisher Scientific. Peptides were separated using a 5 cm of 100 µm inner diameter microcapillary trapping column packed with C18 Reprosil resin (paricle size: 5 µm, pore size: 100 Å) from Dr. Maisch GmbH (Ammerbuch, Germany) and an analytical column uPAC Column 50 cm from PharmaFluidics (ESI Source Solutions, Woburn, MA, USA). Separation was achieved by applying a gradient from 5–27% acetonitrile in 0.1% formic acid over 180 min at a flow rate of 200 nl/min. Electrospray ionization was enabled by applying a voltage of 1.8 kV and sprayed from fused silica pico tips (New Objective, Littleton, MA, USA). The mass spectrometer was operated in the data-dependent mode, with a survey scan performed in the Orbitrap in the range of 395–1,800 m/z at a resolution of 6 × 10^4^, followed by CID-MS/MS fragmentation of the 20 most intense ions in the ion trap, using a precursor isolation width window of m/z 2, AGC setting of 10,000, and a maximum ion accumulation of 200 ms. Singly charged ion species were not subjected to CID fragmentation. The normalized collision energy was set to 35 V and an activation time of 10 ms. Ions within a 10 ppm window around ions selected for MS/MS were excluded from further selection for fragmentation for 60 s.

### Mass spectrometry data analysis

Raw data were analyzed using Proteome Discoverer 2.4 software (Thermo Fisher Scientific). MS/MS spectra were assigned using the Sequest HT algorithm by searching against a protein sequence database including known contaminants and a user-submitted Amyloid database. Sequest HT searches were performed with a 20 ppm precursor ion tolerance and allowing up to two missed cleavages. A 1% false discovery rate (FDR) on both protein and peptide levels was achieved by applying a target-decoy database search. Filtering was done using Percolator and Carbamidomethyl and methionine oxidation were set as static and variable modifications respectively. Label-free quantification (LFQ) of proteins was conducted using LFQ functions of Proteome Discoverer 2.4. Identified proteins were analyzed for pathway enrichment using DAVID Bioinformatics Resources 6.8.

### Site-specific eIF2α mutagenesis

To generate expression constructs harboring the mutated eIF2α O-GlcNAcylation or phosphorylation sites, site-directed mutagenesis was performed using the Q5^®^ Hot Start High-Fidelity system (NEB, Ipswich, MA, USA), according to the manufacturer’s instruction, with primers designed to generate the desired mutations (sequences listed in Additional file 2: Table S3). Briefly, after PCR amplification, the resulting PCR products were directly added to Kinase-Ligase-Dpn1 (KLD Enzyme Mix) for 5–10 min to circularize the PCR products and to remove the template. The ligated PCR products were transformed into NEB® 5-alpha Competent *Escherichia. coli* (High Efficiency) (NEB) following manufacturer’s instruction. The bacterial cells were selected using LB agar plates (Sigma-Aldrich) containing either ampicillin (50 μg/ml; Thermo Fisher Scientific) or kanamycin (50 μg/ml; Thermo Fisher Scientific) at 37 °C overnight. Single colonies were selected and subjected to plasmid purification using the QIAprep Spin Miniprep Kit (Qiagen, Hilden, Germany) following manufacturer’s protocol. Purified plasmid DNAs were sequenced by Integrative Genomics Core (City of Hope, Duarte, CA, USA) for the final identification of the desired clones.

### Overexpression or knockdown transfection

DNA (2 μg) of HO-1 expression construct pcDNA3.1 + *HMOX1*/C-(K)DYK (GenScript, Piscataway, NJ, USA) or eIF2α expression construct pcDNA3.1 + *EIF2S1*/C-(K)DYK (GenScript) was mixed with Lipofectamine 2000 (4 μl; Thermo Fisher Scientific) and transfected into 5 × 10^5^ cells (per well/6-well plate) for HO-1 or eIF2α transient overexpression, according to manufacturer’s instruction. At 48 h post-transfection, the expression of the transfected protein of interest was validated by immunoblot analysis. Individual siRNA targeting HO-1 (Sigma-Aldrich, St. Louis, MO, USA), and eIF2α (Sigma-Aldrich) was used for respective knockdown, respectively. Briefly, 5 × 10^5^ cells per well were seeded into a 6-well plate. Individual siRNAs (30–40 nM) mixed with Lipofectamine RNAiMAX (90–120 μl; Thermo Fisher Scientific) were used for transfection per well to achieve transient knockdown of protein of interest. At 48 h post-transfection, the expression of protein of interest was validated by immunoblot analysis.

### Lentiviral production

3.4 × 10^6^ HEK293T cells were seeded into a 10-cm dish at least 24 h prior to the day of transfection. Once the cells were attached to the plate, the existing medium was replaced with fresh DMEM (10 ml; Thermo Fisher Scientific). The transfection mixture was prepared as follows: Lipofectamine 2000 (51 μl; Thermo Fisher Scientific) was mixed with the psPAX2 DNA (5.6 μg, the viral packing vector; Addgene, Watertown, MA, USA), pMD2.G DNA (5.6 μg, the viral envelope vector; Addgene), and eIF2α lentiviral plasmid DNA (pReceiver-Lv203EIF2SA/C-FLAG-SV40-eGFP-IRES-puromycin; 5.6 μg). Lipofectamine™ 2000 (51 μl) and plasmid DNAs were added to Opti-MEM (850 μl; Thermo Fisher Scientific) and then mixed at room temperature for 5 min. The Lipofectamine/DNA mixture was then added to the HEK293T cells and allowed to incubate for 24 h. On the following day, the medium was aspirated, and fresh DMEM (8 ml) was added to the cells, which were allowed to incubate for another 24 h. The virus particles were harvested at 48, 72, and 96 h post-transfection and pooled. The resulting viral stock was aliquoted and stored at − 80 °C.

### Stable cell line generation

Stable cell lines were generated from transfection or viral transduction of desired protein followed by antibiotic selection. To generate HO-1 stably overexpressing cell line, pcDNA3.1 + *HMOX1*/C-(K)DYK DNA (2 μg/well) was transfected, using Lipofectamine 2000, into 5 × 10^5^ cells/well. At 48 h post-transfection, G418 (1 μg/ml; MilliporeSigma) was added for selection. After a one-week selection, HO-1 protein expression was analyzed by immunoblotting analysis using antibodies against HO-1 (Novus Biologicals, Centennial, CO, USA) and FLAG (Proteintech, Rosemont, IL, USA), respectively. To generate eIF2α stably overexpressing cell line, 500–1000 μl of lentivirus particles based on pReceiver-Lv203EIF2S1/C-Flag-SV40-eGFP-IRES-puromycin (GeneCopoeia, Rockville, MD, USA) were used to transduce 5 × 10^5^ cells in the presence of polybrene (10 µg/ml; MilliporeSigma). Transduction efficiency was monitored by green fluorescent protein (GFP) after 24 h infection. The virus-containing medium was replaced with the complete medium and then puromycin (1 μg/ml; MilliporeSigma) was added for selection. eIF2α protein expression was confirmed by immunoblotting analysis.

### RNA isolation and quantitative real time polymerase chain reaction (qPCR)

TRIzol® Reagent (Invitrogen, Waltham, MA, USA) was used for RNA isolation following manufacturer's protocol [45]. The RNA pellet was resuspended in RNase-free water (30–50 µl). To quantify the target mRNA levels, RNA was first reversed transcribed into cDNA, using the iScript™ cDNA synthesis kit (BioRAD, Hercules, CA, USA) following manufacturer's protocol. The cDNA was then subjected to CFX96™ Real-Time PCR detection system (BioRAD) with iTaq Universal SYBR Green Supermix (BioRAD) according to the manufacturer’s instruction. The reaction was performed in the iQ5 Thermal Cycler (Bio-Rad) and data was analyzed by the 2^−ΔΔCt^ method [46] and normalized to *GAPDH*; n = 3. The primer sequences are listed in Additional file 2: Table S1.

### Reactive oxygen species (ROS) analysis

Cells were incubated with 2′-7′-dichlorodihydrofluorescein diacetate (DCF-DA; 1 μM; MilliporeSigma) or CellROX™ Deep Red Reagent (Invitrogen) in the culture medium for 30 min. Cells were collected and resuspended in PBS (1 ml) containing BSA (1%), and analyzed immediately by flow cytometer Accuri C6 (BD, Franklin Lakes, NJ, USA) using the FL-1 green or FL-4 red fluorescence detectors as described previously [7, 33].

### Cell viability and cell recovery assay

Cell viability was determined by using the acid phosphatase (ACP) assay. Briefly, cells were plated in 12-well plates and incubated for defined periods based on experimental design. The cells were then washed with PBS once and incubated with ACP assay buffer (400 μl, 0.1 M sodium acetate and 0.1% Triton X-100, pH 5.0), supplemented with 4-Nitrophenyl phosphate disodium salt hexahydrate (pNPP) (7 mM; MilliporeSigma) at 37 °C for 30 min. After incubation, NaOH (40 µl, 1N) was added to each well. Absorbance at 410 nm was measured with a SYNERGY H1 microplate reader (BioTEK, Winooski, VT, USA). The cell recovery assay was performed as a modified cell viability assay. Briefly, following treatment, such as Arg withdrawal for defined periods, the culture medium was replaced with a complete medium and incubated for an additional 24, 48, or 72 h, prior to ACP assays, depending on the experiment design.

### Cell migration assay

Cells were suspended in 1 × 10^5^ cells/ml mixture with culture medium, and the cell mixture (70 μl) was then seeded into the Culture-Insert 2-Well (iBiDi, Gräfelfing, Germany). After overnight incubation to allow cell attachment, the 2 well silicon insert was removed to create a cell-free gap. The plates were gently filled with the desired medium to allow for the visualization of cell migration.

### Sphere formation, cryosection, and immunofluorescence

5–8 × 10^3^ BT-549 cells were seeded into 96-cell Ultra-Low Attachment plates (Corning Incorporated, NY, USA) and incubated in the complete medium for 48 h to allow for sphere formation. Then the complete medium was replaced with Arg-free medium for the next 24 h. Spheres were collected for immunofluorescent staining analysis. Briefly, spheres were fixed in paraformaldehyde (4%) at 4 °C overnight. After washing with PBS three times, spheres were embedded in a cryomold (Thomas Scientific, Swedesboro, NJ, USA) with Tissue-Tek^®^ Optimal Cutting Temperature (O.C.T.) (Sakura Finetek, Torrance, CA, USA) compound. Embedded cell spheres can be stored at − 80 °C or subjected to cryosection to slice an 8-μm thick sphere section using Research Cryostat (Leica Biosystems, Wetzlar, Germany). Sphere sections were fixed on X-tra clipped adhesive microscope slides (Leica Biosystem) for the immunofluorescent staining. Briefly, sphere sections were incubated/treated in Triton X-100 (0.1% in PBS) for 15 min to permeabilize the cells. The samples were blocked with BSA/Triton X-100 (2% BSA and 0.3% Triton X-100 in PBS) for 1 h. Sections were then incubated with the primary anti-HO-1 antibody (1:200 dilution). in a 4 °C humidified incubator overnight. After the unbound primary antibodies were removed by washing three times with PBS, the sphere section was incubated with a goat anti-mouse secondary antibody conjugated with Alexa Fluor 488 (1:500 dilution; Thermo Fisher Scientific) at room temperature for 1 h. The sections were then incubated in DAPI solution (5 μM; Invitrogen) for 10 min to localize the nucleus, followed by two PBS washes. Sections were dried and mounted in ProLong Diamond Antifade Mountant (Invitrogen) for permanent storage. Fluorescent mages were captured by a Zeiss Observer II microscope (Zeiss, Jena, Germany). HO-1 signals were quantified by Image J (NIH, Bethesda, MD, USA).

### *HMOX1* ARE-driven reporter assay

The dual firefly and Renilla reporter assay was performed to test *HMOX1* antioxidant response element (ARE)-dependent transcriptional control. Briefly, cells were seeded into 12-well plates at a density of 2 × 10^5^ per well and incubated overnight. Cells were then transiently co-transfected with *HMOX-1* ARE-FLuc reporter plasmid (from Reen Wu, UC Davis, CA, USA) or firefly luciferase empty vector, as well as pRL-SV40 Renilla luciferase reporter plasmid (Promega, Madison, WI, USA) (serving as an internal control). At 48 h post-transfection, cells were transferred to Arg-free medium for an additional 24 h prior to dual luciferase assay. *HMOX-1* ARE-FLuc firefly and Renilla luciferase activities were assessed by adding ONE-Glo™ EX Reagent and NanoDLR™ Stop & Glo® Reagent sequentially using Dual-Luciferase^®^ Reporter Assay System according to manufacturer’s instruction (Promega). Firefly luminescence and *Renilla* luminescence were measured by SYNERGY H1 microplate reader. All the firefly luminescence readings were normalized with Renilla luminescence values and presented as the relative fold differences compared to the control group.

### Metabolic labeling of newly synthesized proteins

The Click-iT Protein labeling method [47] was used to label newly synthesized proteins. Cells were incubated in Met-free medium (Axxora, Farmingdale, NY, USA) containing the Met analog l-azidohomoalanine (AHA; 250 μM; Click Chemistry Tools, Scottsdale, AZ, USA) for 6 h. After incubation, cells were lysed in RIPA buffer (300 μl, 50 mM Tris HCl, pH 7.4, 150 mM NaCl, 2.5 mM Tris (2-carboxyethyl)phosphine) and PhosSTOP™ phosphatase inhibitor cocktail (1X). After centrifugation at 12,000 ×*g* at 4 °C for 20 min, the supernatant was collected. 1 mg of protein supernatant was used for pull-down assay. Approximately 1 mg equivalent of protein supernatant was used for pull-down assay by adjusting the volume to 300 μl with RIPA buffer. The newly synthesized AHA-incorporated protein was crosslinked to biotin-PEG4-alkyne by a Cu(I) (ACROS, Waltham, MA, USA)-catalyzed cycloaddition (Click-iT) reaction. The reaction mixture (200 μl/1 mg protein sample, 0.2 mM biotin-PEG4-alkyne, 1 mM L-ascorbate, 1 mM CuSO4, and 0.1 mM Tris (3-hydroxypropyltriazolylmethyl)amine ligand) was prepared in potassium phosphate buffer (200 μl; 100 mM, pH 7.0) and the unconjugated biotin-PEG4-alkyne reagent was removed by chloroform/methanol participation as described above. The resulting protein disk was washed with methanol (1 ml) two times and resuspended in resuspension buffer (200 μl; 50 mM 4-(2-hydroxyethyl)-1-piperazineethanesulfonic acid, 2% SDS, 150 mM NaCl) with briefly sonicated. The protein was diluted with 600 μl of RIPA buffer, and 30 μl of a 50% Streptavidin Agarose slurry was added to the sample. The samples were incubated on a rotator overnight at room temperature. The beads were centrifuged at 500 ×*g* for 2 min, followed by six washes with 1 mL of RIPA buffer and two washes with ddH_2_O. The pulled-downed proteins were eluted into sample buffer by boiling for 15 min. Total protein inputs and newly synthesized proteins that were pulled-down were analyzed by immunoblotting.

### Gene expression and overall survival analysis

*HMOX-1* gene expression data from the TCGA (The Cancer Genome Atlas) Pan-Cancer project [48] was used in this study. Log2-transformed RNA-Seq by Expectation–Maximization (RSEM) expression values were used for boxplots. Statistical p-values between groups were determined by Wilcoxon tests. The data from Tang et al. [49] was used to plot the Kaplan–Meier curves for estimating overall survival, including 58 breast tumors having both HO-1 proteomics and survival data with the time-to-death greater than 0. The group with detectable HO-1 (peptide spectral counts larger than zero; n = 16) was compared to the group with no detectable HO-1 peptides (n = 42). The analysis was conducted using survival package (version 3.2.10) in R.

### Xenograft mouse model, Arg-free diet, and tumor characterization

Luciferase-labeled BT-549 cells (3.3 × 10^5^) were injected into the mammary fat pads of 6-week-old female NOD.Cg-PrkdcscidIl2rgtm1Wjl/SzJ (NSG) mice as reported in [7, 33, 35, 36]. The recipient mice were then divided into 2 groups. One group was fed an Arg-free diet (Teklad diet TD.09152; ENVIGO, Indianapolis, IN, USA), and the other group was fed a control diet (Teklad diet TD.01804; ENVIGO). The diet feeding was a week prior to tumor implantation and continued for 3 weeks. At 2 weeks post tumor implantation, the tumor masses were excised *en bloc* and then fixed in 10% neutral-buffered formalin for 48 h.

### Immunohistochemistry (IHC) analysis

The tissue samples were processed by the Pathology Core at City of Hope, which included embedding, sectioning, and immunohistochemical staining. The HO-1 IHC analysis was performed using the Ventana Discovery Ultra (Ventana Medical Systems, Roche Diagnostics, Indianapolis, USA) automated stainer. Briefly, 5 μm sections of tissue were mounted on positively charged glass slides. The slides were deparaffinized and rehydrated, then treated with an endogenous peroxidase activity inhibitor and antigen retrieval reagent. They were then incubated with an anti-human HO-1 mouse monoclonal primary antibody (1:500; Novus Biologicals), followed by an anti-mouse IgG1 + IgG2a-IgG3 rabbit monoclonal secondary antibody (1:500; Abcam, Cambridge, UK). The staining was visualized using the ChromoMap DAB Kit (CRB DISCOVERY) and counterstained with hematoxylin (Ventana) and cover slips. After IHC staining, whole slide images were acquired using the NanoZoomer S360 Digital Slide Scanner (Hamamatsu Photonics, Las Vegas, NV, USA) and viewed using the NDP.view image viewer software (Hamamatsu Photonics).

### Statistical analysis

Data are presented as the mean and standard error of the mean (mean ± s.e.m.). Statistical analyses were conducted using GraphPad Prism 7.0 software (GraphPad Prism Software Inc., San Diego, CA, USA). Most experiments included at least three independent biological replicates for each condition. Two Way ANOVA-Tukey or Dunnett multiple comparison and One Way ANOVA were used to compare means. The center value is defined as the mean value, and s.e.m. was used to calculate and plot error bars from raw data. For TCGA RNA-Seq database analyses with large sample sizes, the non-parametric Wilcoxon test was used. A *p*-value of < 0.05 was considered statistically significant.

## Results

### ArgS alters O-GlcNAcylation of translational factors

To examine whether Arg regulates protein O-GlcNAcylation, we first examined global protein O-GlcNAcylation profiles in the ± Arg contexts (Additional file 1: Fig. S1A). As a positive control, we confirmed that BT-549 breast cancer cells exhibiting Glc- and Gln-sensitive (Additional file 1: Fig. S1A). Immunoblotting blot analysis with an O-GlcNAc-specific antibody revealed that global O-GlcNAcylation was suppressed by ArgS (Additional file 1: Fig. S1A, 3rd panel). After confirming that ArgS, like other metabolic stress induced by Glc or Gln deprivation, repressed the global O-GlcNAcylation profile, we sought to understand how O-GlcNAcylation is impacted by nutrient restriction. Given that O-GlcNAcylation can be regulated by the addition or removal of UDP-GlcNAc to target proteins, we therefore tested whether OGT and OGA levels were sensitive to nutrient restriction in BT-549 cells. We found that no consistent changes in OGT or OGA level were observed by Western analyses under Glu, Gln or Arg withdrawal (Additional file 1: Fig. S1A, top 2 panel). The level of UDP-GlcNAc, a substrate for OGT, is controlled by the enzymes in HBP pathway (Additional file 1: Fig. S1B). To address the possibility that ArgS regulates O-GlcNAcylation at the level of UDP-GlcNAc generation, we explored whether ArgS-mediated suppression of O-GlcNAcylation is associated with the alterations in the gene expression of the HBP. Our RT-PCR analysis; however, demonstrated an increase in the mRNA expression of all HBP enzymes, including the rate-limiting enzyme glutamine-fructose-6-phosphate aminotransferase 1 (GFAT1) during ArgS (Additional file 1: Fig. S1C). It is possible that ArgS does not suppress global O-GlcNAcylation at the level of O-GlcNAc cycling enzymes or its source.

**Fig. 1:**
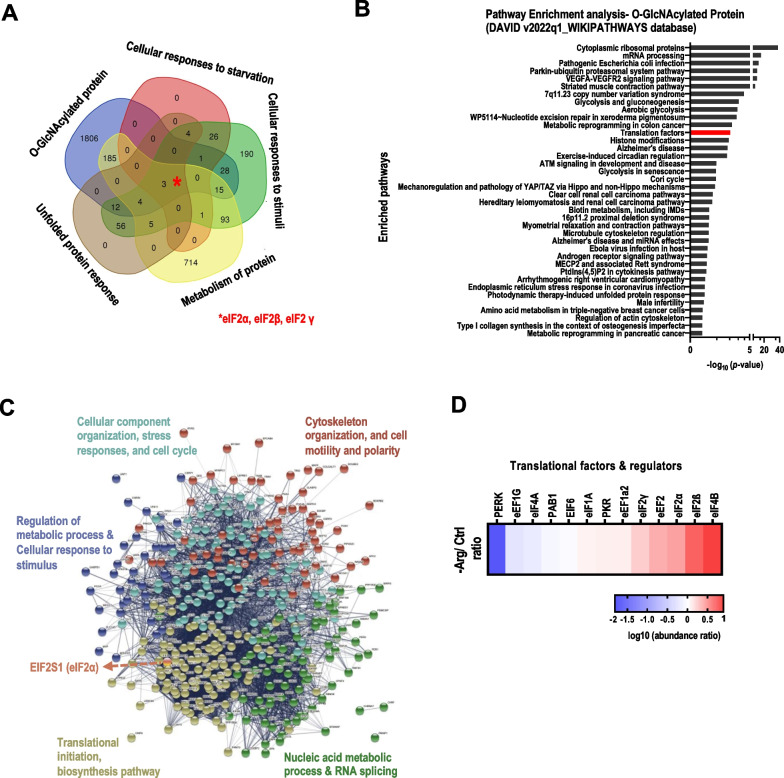
The dynamic changes of eIF2 O-GlcNAcylation upon ArgS. **A** A Venn diagram was generated to depict the overlap between 2054 O-GlcNAz-modified proteins that were pulled down (Additional file 3: Table S4, S5) in different ArgS-induced pathways (as obtained from Reactome.org). **B** A pathway enrichment analysis was performed on O-GlcNAz-modified proteins (using the WIKIPATHWAYS database) to identify those significantly affected (FDR < 0.05) by ArgS. **C** The 363 O-GlcNAz-modified proteins identified in the pathway enrichment analysis **B** were grouped by function using the STRING database. **D** A heatmap was generated to show the log10 abundance ratio of O-GlcNAz-modified proteins involved in eukaryotic protein translation that were significantly changed (FDR < 0.05) based on proteomics analysis under the indicated conditions. The O-GlcNAz-modified eukaryotic factors/regulators were clustered based on their level changes relative to control, with samples prepared from BT-549 cells (n = 3). The cells were treated with an -Arg medium (or not) and GlcNAz (50 μM) for 48 h prior to collection and protein extraction

Next, we utilized GlcNAz-based proteomics (Additional file 1: Fig. S1D) to identify 2054 O-GlcNAz-modified proteins, whose levels were influenced by ArgS in BT-549 cells. While the majority of identified O-GlcNAz-modified proteins showed decreased abundance as expected, a minor fraction of O-GlcNAz-modified proteins increased (Additional file 1: Fig. S1E), revealing both positive and negative effects on the protein O-GlcNAcylation landscape. We speculate that the decreased overall O-GlcNAcylation upon ArgS (Additional file 1: Fig. S1A) might reflect relatively higher overall abundance for these down-regulated proteins in BT-549 cells. Of the 2054 O-GlcNAz-modified proteins identified, 248 O-GlcNAz-modified proteins were detected in 4 major pathways: cellular response to starvation, unfolded protein response (UPR), cellular responses to stimuli (hypoxia, reactive oxygen species, and heat), and metabolism of proteins (Fig. 1A, Additional file 3: Table S4, S5). Notably, 3 of enriched 248 O-GlcNAz-modified proteins, EIF2S1 (eIF2α), EIF2S2 (eIF2β), and EIF2S3 (eIF2γ), were associated with all 4 ArgS-regulated pathways (Fig. 1A). To gain more insight into the impact of ArgS-regulated O-GlcNAcylation, DAVID bioinformatics database was queried to visualize functional pathways partaken by these 2054 O-GlcNAz-modified proteins in parallel. Figure 1B, C show that 363 of 2054 O-GlcNAz-modified proteins were placed into 38 functional pathways, including cytoplasmic ribosomal proteins, translational initiation, and biosynthesis pathway. Again, 13 out of 363 O-GlcNAz-modified proteins, including eIF2α, were related to eukaryotic protein translation and/or eIF2 regulation (Fig. 1D), raising the question of what role eIF2α O-GlcNAcylation might play in the adaptive response to ArgS.

### eIF2α O-GlcNAcylation downregulates antioxidant capacity, cell recovery and migratory ability upon ArgS

**Fig. 2:**
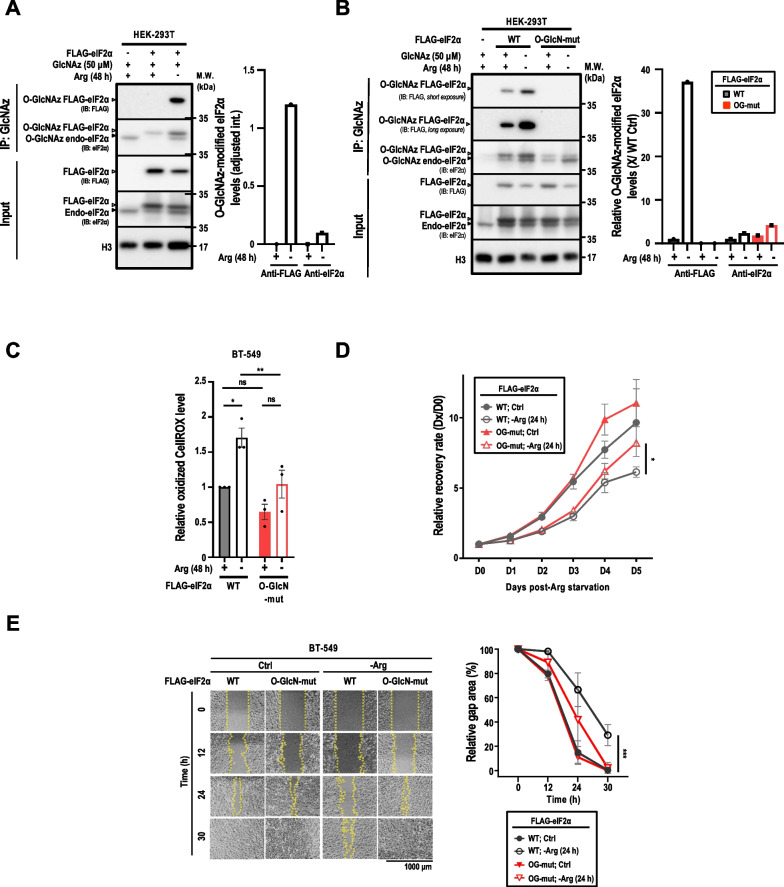
The O-GlcNAcylation of eIF2α downregulates the antioxidant capacity, cell recovery and migratory ability of breast cancer cells in response to ArgS. **A** Immunoprecipitation coupled with immunoblot analysis was performed to measure the levels of GlcNAz-modified (O-GlcNAcylated) proteins in parental HEK 293T cells (n = 3; *left panel*). The O-GlcNAz-modified eIF2α level was calculated by normalizing the densitometric tracing of eIF2α signal to the H3 signal (*right panel*). The values were quantified using ImageLAB software and adjusted for background. **B** The same analysis was performed in WT and O-GlcN-mut, eIF2α overexpressing HEK 293 T cells (n = 2; *left panel*). The O-GlcNAz-modified eIF2α level was calculated as described in **A**, and the ratio in each experimental condition was compared to the reference (WT + Arg, set to 1; *left panel*). One representative immunoblot is shown for each analysis, with indicated antibodies. The black arrowheads indicate endogenous eIF2α, and the white arrowheads indicate FLAG-tagged eIF2α. **C** The levels of ROS were measured in BT-549 cells stably expressing WT or O-GlcN-mut eIF2α after 48 h of ArgS treatment (n = 3). The ROS levels were quantified using CellROX™ and flow cytometry. **D** The relative cell recovery was monitored in BT-549 cells stably overexpressing WT or O-GlcN-mut eIF2α over a 5-day period, after 24 h of ArgS treatment and replacement with complete medium. The cell recovery was determined using ACP assays and the data were normalized to the values of Day 0, set to 1. **E** The cell migration was measured in BT-549 cells stably overexpressing WT or O-GlcN-mut eIF2α after 24 h of ArgS treatment and replacement with complete medium. The cell migration was determined by calculating the % confluency within the gap area and was imaged and quantified using Image J software at 0, 12, 24, and 30 h. Data are presented as mean ± s.e.m. and analyzed using Two-Way ANOVA followed by Tukey's multiple comparison test. Statistical significance was determined with *p < 0.05; **p < 0.01; ***p < 0.001

Based on the aforementioned data, we hypothesized that ArgS could regulate protein translational initiation by inducing eIF2α O-GlcNAcylation. This can help determine whether it is an adaptive response pro-survival or not. To test this possibility, we utilized a metabolic labeling approach with GlcNAz and azide-phosphine (Staudinger) ligation coupling strategies (Additional file 1: Fig. S1D). We confirmed that HEK293T cells deprived of Arg displayed heightened signals of O-GlcNAz-modified endogenous eIF2α, as well as exogenous FLAG-tagged eIF2α, within enriched O-GlcNAz-modified proteins compared to control cells (Fig. 2A). To further examine the role of eIF2α O-GlcNAcylation in this process, we designed an O-GlcN-mut eIF2α expression construct by replacing Ser219, Thr239, and Thr241 [50] with alanine (Ala) via site-specific mutagenesis. Using pulldown followed by Western analysis, we tested whether the Ser219, Thr239, and Thr241 residues are the major ArgS-induced O-GlcNAcylation sites on eIF2α. Specifically, an anti-FLAG antibody was used to detect the FLAG-tagged O-GlcN-mut eIF2α in pulled-down O-GlcNAz-modified proteins under both control (complete medium) and ArgS conditions. Of note, even with a longer exposure (Fig. 2B, 2nd panel), we did not detect any O-GlcNAcylation of the mutant eIF2α, confirming that these three residues are indeed the major ArgS-induced O-GlcNAcylation sites on eIF2α. Next, we examined whether eIF2α O-GlcNAcylation was sensitive to other nutrient stresses in BT-549 cells. Specifically, we tested the effects of Gln and Glc withdrawal on endogenous eIF2α O-GlcNAcylation levels using O-GlcNAz-modified eIF2α as a marker. Interestingly, we found that unlike ArgS, neither Gln restriction nor Glc limitation was able to increase O-GlcNAz-modified eIF2α levels (Additional file 1: Fig. S1F). These results suggest that ArgS is a unique metabolic stressor that induces eIF2α O-GlcNAcylation in BT-549 cells under different nutrient stress conditions.

Lastly, to assess the functional impact of the loss of eIF2α O-GlcNAcylation on the adaptive response to ArgS, assays for oxidative stress, cell recovery from ArgS, and cell migration were performed. As shown in Fig. 2C, overexpression of O-GlcN-mut eIF2α nearly abolished the increase in ArgS-induced oxidized CellROX signals in BT-549 cells. Next, we developed a cell recovery assay to evaluate the effect of eIF2α O-GlcNAcylation on cell recovery from ArgS. The cell recovery assay is based on the daily cell viability after Arg is re-supplemented at 24 h post ArgS for 5 days. An improved cell recovery following ArgS was noticed in O-GlcN-mut eIF2α-overexpressing BT-549 cells, compared with wild type (WT) eIF2α (Fig. 2D). Lastly, O-GlcN-mut eIF2α conveyed improved cell migration upon ArgS (Fig. 2E). We thereby surmised that ArgS induces eIF2α O-GlcNAcylation leading to impaired cell recovery and migration and elevated ROS accumulation.

### ArgS downregulates HO-1 protein level to promote oxidative stress

**Fig. 3:**
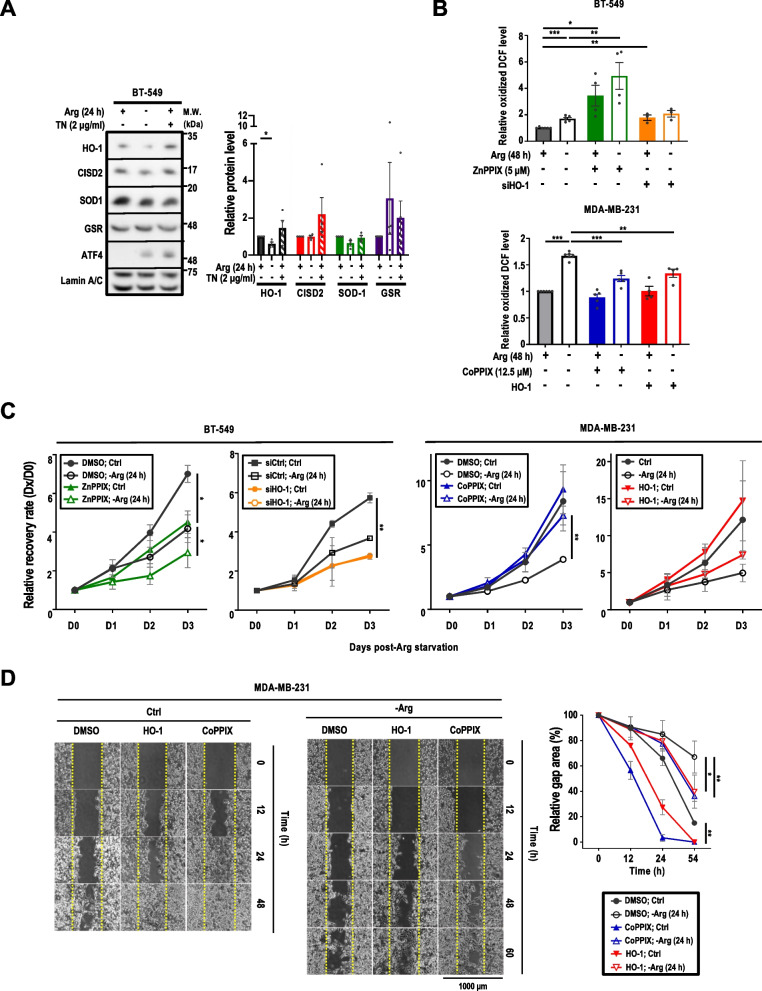
ArgS regulates antioxidative proteins to decrease cell recovery and migration. **A** Immunoblot analysis (*left panel*) of the antioxidant proteins HO-1, CISD2, SOD1, and GSR in BT-549 cells treated with ArgS or TN (2 μg/ml) for 24 h. One representative immunoblot (n = 3) is shown. The relative protein level (*right panel*) was calculated by normalizing the protein signal intensity in the + Arg group, which was set to 1 after normalization with Lamin A/C. **B** Analysis of ROS levels in BT-549 (*top panel*) and MDA-MB-231 (*botto*m *panel)* cells treated with a combination of ArgS and HO-1 activators or inhibitors (CoPPIX or ZnPPIX) or HO-1 knockdown/overexpression. BT-549 cells were transfected with *siHMOX1* (30 nM) or treated with ZnPPIX (5 μM), and MDA-MB-231 cells were stably overexpressing HO-1 or treated with COPPIX (12.5 μM). ROS levels were monitored by DCF-DA oxidation after 24 h of ArgS treatment. **C** Analysis of cell recovery in BT-549 (*left panel*) and MDA-MB-231 (*right panel*) cells treated with a combination of ArgS and HO-1 activators or inhibitors (CoPPIX or ZnPPIX) or HO-1 knockdown/overexpression, as described in **B**. The cell recovery was determined as described previously in Fig. 2D. **D** Analysis of cell migration in MDA-MB-231 cells stably overexpressing HO-1 or treated with COPPIX (12.5 μM) after 24 h of ArgS treatment, as described previously in Fig. 2E; n = 3. Data are shown as mean ± s.e.m.; *p < 0.05; **p < 0.01; ***p < 0.001; Two-Way ANOVA followed by Dunett’s (**A**) or Tukey's (**B**-**D**) multiple comparison tests

To unravel the mechanism underlying how eIF2α O-GlcNAcylation promotes oxidative stress in Arg-starved cells, we investigated the expression level of selected antioxidant defense and redox-regulatory proteins, namely the HO-1, CDGSH iron-sulfur domain-containing protein 2 (CISD2), superoxide dismutase type 1 (SOD-1), and glutathione reductase (GSR) [40, 51–53]. We have previously shown that ArgS-triggered UPR induces transcriptional factor ATF4 expression [7]. ATF4 regulates the expression of genes involved in the antioxidant protein synthesis to reduce oxidative stress. Here, we compared these antioxidant protein levels in the ± Arg context to those of cells treated with a known ER stress inducer, TN (2 μg/ml) [41]. Compared to the control (complete medium) and TN treatment, ArgS reduced HO-1 expression (Fig. 3A). Likewise, treatment with ADI-PEG20 (1 μg/ml), which hydrolyzes Arg to citrulline and ammonia [44], also significantly decreased HO-1 protein levels (Additional file 1: Fig. S2A), suggesting that our observations on HO-1 downregulation applied to both ArgS (by removing extracellular Arg) and Arg deprivation (using ADI-PEG20). This downregulation of HO-1 protein level upon ArgS was reversible by Arg replenishment, reaching the max at 12 h post Arg re-supplementation (Additional file 1: Fig. S2B). The observed downregulation of HO-1 and SOD-1 distinguished ArgS from the canonical ER stress. Next, TCGA database indicated that all mRNAs encoding HO-1, CISD2, SOD-1 and GSR in breast tumors were significantly higher than those in normal tissues (Additional file 1: Fig. S2C [48]). It is worth noting that *HMOX1* mRNA, which encodes HO-1, was significantly higher in tumor tissues than in their corresponding normal tissues in 5 out of 17 common cancer types, including breast cancer (Additional file 1: Fig. S2D [48]). In addition, *HMOX1* mRNA abundances in primary breast tumors of all 4 molecular subtypes were higher than in normal breast tissues (Additional file 1: Fig. S2E [48],). Lastly, Kaplan–Meier survival curves revealed that HO-1 protein levels in tumor were inversely correlated with the overall survival rate in breast cancer patients (Additional file 1: Fig. S2F [49],). Based on these observations, we hypothesized that the downregulation of HO-1 resulted in the elevated ROS accumulation upon ArgS. To test this possibility, we first examined HO-1 protein abundance in eight different breast cancer cells and found that BT-549 expressed high levels of HO1 whereas MDA-MB-231 expressed nearly undetectable levels of HO-1 (Additional file 1: Fig. S3A). To ascertain the relationship between HO-1 expression/activity and the cell fate in the context of ArgS, we overexpressed and knocked down HO-1 or induced and inhibited HO-1 activity in HO-1 low MDA-MB-231 cells and HO-1 high BT-549 cells, respectively. As such, BT-549 cells were transfected with *siHMOX1* or treated with an HO-1 competitive inhibitor, ZnPPIX (5 μM), an analog of heme [17, 18, 54], to suppress HO-1 abundance or activity (Additional file 1: Fig. S3B*, *left panels). Consistent with previous reports [17, 18, 54], HO-1 protein expression was induced in ZnPPIX-treated BT-549 cells (Additional file 1: Fig. S3B, *lower left panel*). Both genetic and pharmacological manipulations notably induced ROS production upon ArgS compared with control (Fig. 3B, upper panel, Additional file 1: Fig. S3C). In contrast, a significant reduction of ArgS-induced ROS was observed in MDA-MB-231 cells stably transfected with HO-1 expression construct or treated with a HO-1 activator, CoPPIX ([39], 12.5 μM) (Fig. 3B, lower panel). These results suggested an inverse relationship between HO-1 and ROS levels.

Upon ArgS (24 or 48 h), the cell viability of BT-549 and MDA-MB-231 cells significantly decreased by more than 50% (Additional file 1: Fig. S3D, left 2 panels). To substantiate that HO-1 is needed to support cell recover following ArgS, the cell viabilities were tested by ACP assay daily after Arg was re-supplemented at 24 h post-ArgS. As shown in Fig. 3C (*left two panels*), both ZnPPIX treatment and HO-1 knockdown delayed BT-549 cells’ recovery from ArgS. On the other hand, treatment with CoPPIX or stably overexpressing HO-1 improved MDA-MB-231 cells’ recovery from ArgS (Fig. 3C, right two panels). Likewise, HO-1 overexpression allowed MDA-MB-231 cells to recover better from treatment with ADI-PEG20 (1 μg/ml, 24 h, Additional file 1: Fig. S3D, right 2 panels). Lastly, cell migration assays showed that ArgS decreased MDA-MB-231 cell migration (Additional file 1: Fig. S3E). CoPPIX treatment or HO-1 overexpression enabled cells to migrate faster under both control and ArgS (Fig. 3D). Together, we conclude that HO-1 expression/activity is necessary and sufficient to enable breast cancer cells to recover and migrate better with reduced ROS in the context of ArgS.

### ArgS induces eIF2α O-GlcNAcylation to downregulate HO-1 protein translation

Amino acid deprivation is known to activate ISR kinase [55] to phosphorylate eIF2α, a critical factor of the ER stress response, at Ser51 to suppress the global translation initiation [7, 8]. To assess how ArgS attenuates HO-1 protein expression, we turned our attention to the ER stress-induced eIF2α Ser51 phosphorylation as a potential mechanism. Co-treatment with TUDCA ([42], 200 and 400 μM), a chemical chaperon attenuating ER stress, rescued HO-1 protein in Arg-starved BT-549 cells (Additional file 1: Fig. S4A). However, co-treatment with ISRIB (400 nM, interrupting the interaction between eIF2α and EIF2B [43], failed to rescue HO-1 protein level under ArgS (Additional file 1: Fig. S4B). Next, to examine whether Ser51-phosphorylated eIF2α (p-eIF2α) attenuated HO-1 protein expression, we engineered a mutation at eIF2α Ser51 to abolish its phosphorylation. However, the Ser51Ala (S51A)-mutated eIF2α (phospho-mut eIF2α) only abolished ATF4 expression but failed to rescue the downregulation of HO-1 protein expression in Arg-starved BT-549 cells (Additional file 1: Fig. S4C). Together, these data indicated that p-eIF2α is not required for ArgS to downregulate HO-1 protein expression.

**Fig. 4:**
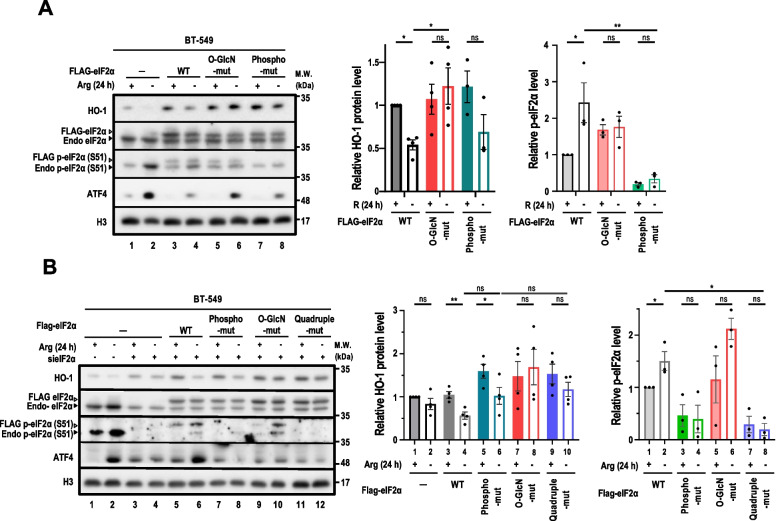
eIF2α O-GlcNAcylation suppresses HO-1 protein expression in Arg-starved breast cancer cells. **A** Immunoblot analysis of HO-1 in BT-549 cells subjected to ArgS for 24 h. One representative immunoblot is shown in the *left panel* (n = 4). BT-549 cells were transiently transfected with FLAG-tagged WT, O-GlcN-mut, or phospho-mut eIF2α. Parental BT-549 cells labeled with (-) serve as a negative control of eIF2α overexpression. The relative levels of HO-1 (*middle panel*) and p-eIF2α (*right panel*) are expressed as fold change and used to compare protein levels across experimental conditions. **B** Immunoblot analysis of HO-1 in BT-549 cells stably WT, phospho-mut, O-GlcN-mut, or quadruple-mutant eIF2α subjected to ArgS for 24 h. One representative immunoblot is shown in the *left panel* (n = 4). Parental BT-549 cells labeled with (-) serve as a negative control of eIF2α overexpression. The relative levels of HO-1 protein (*middle panel*) and p-eIF2α (*right panel*) are expressed as fold change and used to compare protein levels across experimental conditions. **A**, **B** Black arrowheads indicate endogenous eIF2α, while white arrowheads indicate FLAG-tagged eIF2α. The relative p-eIF2α level is calculated by normalizing the densitometric tracing of the p-eIF2α signal with the total eIF2α signal. The ratio in each experimental condition is then compared to the reference (parental; + Arg; set as 1). The relative HO-1 protein level is determined by comparing the densitometric tracing of HO-1 signal in experimental conditions to the reference, with the values of the reference HO-1 and p-eIF2α set as 1 after normalization with H3 (used as a loading control). Data are presented as mean ± standard error of the mean (s.e.m.); *: *p* < 0.05; **: *p* < 0.01; ***: *p* < 0.001. Statistical analysis was performed using Two-Way ANOVA followed by Dunnett's multiple comparison test

Next, we investigated whether eIF2α O-GlcNAcylation affected HO-1 protein expression in the context of ArgS. To achieve this goal, BT-549 cells were transiently transfected with either WT, phospho-mut or O-GlcN-mut eIF2α, and then subjected them to ArgS. Indeed, overexpression of O-GlcN-mut, but not phospho-mut, eIF2α rescued HO-1 in Arg-starved cells (Fig. 4A). To overcome the interference from endogenous eIF2α on HO-1 expression under ArgS, siRNA targeting *eIF2α* 3′-untranslated region (UTR) was used to knock down the endogenous eIF2α. *sieIF2α (3′-UTR)* effectively reduced endogenous eIF2α protein levels (Fig. 4B, Additional file 1: Fig. S4D)*.* However, we noted that knockdown of endogenous eIF2α also caused cell stress, evidenced by the increased ATF4 even in complete medium (Fig. 4B, Additional file 1: Fig. S4D), as reported by others [56, 57]. To assess whether eIF2α Ser51 phosphorylation was involved in ArgS-impaired HO-1 expression, an eIF2α expressing construct in which all three O-GlcNAcylation sites (Ser219, Thr239, and Thr241) and phosphorylation site (Ser51) were substituted with Ala to disrupt O-GlcNAcylation and phosphorylation simultaneously. It appears that the p-eIF2α (or not) did not affect the ability of eIF2α O-GlcN-mut to rescue HO-1 abundance upon ArgS (Fig. 4B, middle panel, lane 8 versus lane 4 and lane 10 versus lane 4). These results suggested that eIF2α O-GlcNAcylation alone plays a key role in suppressing HO-1 protein expression upon ArgS.

**Fig. 5:**
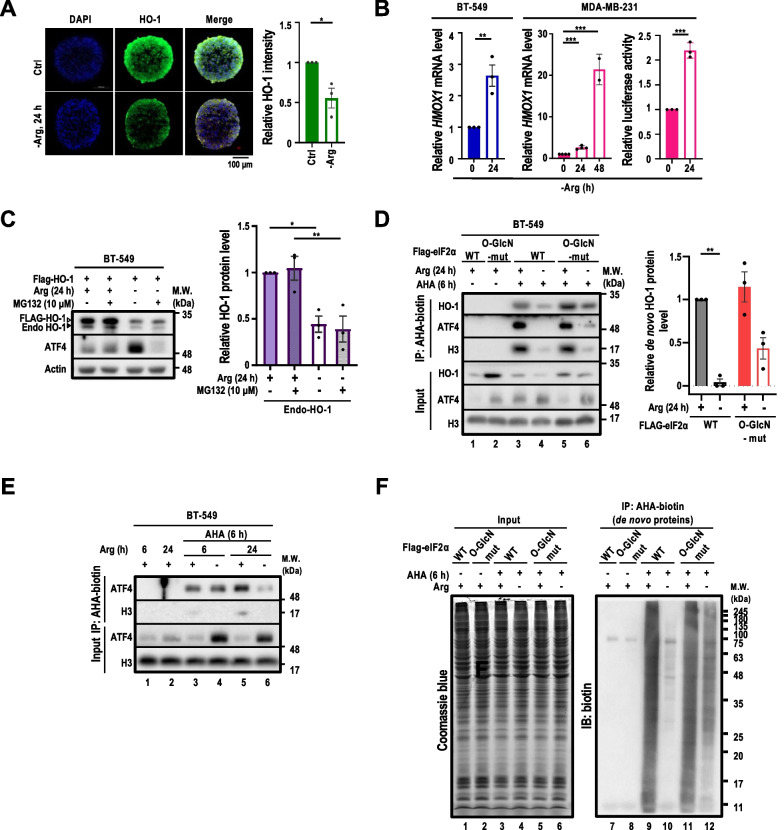
O-GlcNAcylation of eIF2α suppresses HO-1 protein translation. **A** Immunofluorescence of HO-1 and DAPI was conducted in BT-549 cell spheres subjected to ArgS for 24 h (n = 3; *left panel*). The relative HO-1 intensity (*right panel*) was calculated by dividing the integrated HO-1 intensities by the selected sphere area. **B** The levels of *HMOX1* mRNA were analyzed by qRT-PCR in BT-549 and MDA-MB-231 cells and the activity of the *HMOX1* promoter was analyzed by bioluminescent reporter assay in MDA-MB-231 cells. The cells were harvested for total RNA extraction or the bioluminescent reporter assay and subjected to ArgS for 24 or 48 h, respectively (n = 3). qRT-PCR was performed using gene-specific primer pairs and analyzed by the 2^−∆∆Ct^ method. ΔCt refers to the difference in cycle threshold values between the target gene and reference gene. In the bioluminescent reporter assay, cells were transiently transfected with the *HMOX1* ARE-Firefly luciferase (FLuc) reporter construct and the pRL-SV40 Renilla luciferase construct. The intensities of firefly luminescence were normalized with the Renilla luminescence intensities and presented as histograms. **C** Immunoblot analysis of HO-1 in BT-549 cells subjected to ArgS ± MG132 (10 μM) for 24 h (*left panel*). Endogenous and FLAG-tagged HO-1 were visualized using an anti-HO-1 antibody. Actin served as a loading control and ATF4 as an indicator of ER stress. The relative HO-1 protein level (*right panel*) was calculated by normalizing with the level in the control (+ Arg, -MG132), which was set as 1 after normalization with actin. **D** Immunoprecipitation coupled with Western blot analysis of de novo HO-1 synthesis upon ArgS in BT-549 cells overexpressing WT or O-GlcN-mut eIF2α (n = 3). The cells were cultured under ± Arg for 24 h and AHA (250 μM) was added for the last 6 h. The de novo synthesized HO-1 protein level was determined by comparing the densitometric tracing of HO-1 signal in the experimental conditions with the reference HO-1 signal (eIF2α WT; + Arg). The value of reference HO-1 was set as 1 after normalization with H3 (serving as a loading control). AHA-labeled proteins were detected using Click-iT chemistry, and the products were purified with streptavidin beads affinity pulldown. **E** Immunoprecipitation coupled with Western blot analysis of de novo ATF4 synthesis upon ArgS in BT-549 cells overexpressing WT or O-GlcN-mut eIF2α. Cells were exposed to Arg or not for 6 or 24 h with the addition of AHA (250 μM) for the last 6 h prior to harvesting. The pulled-down de novo AHA-labeled ATF4 proteins were then visualized through immunoblotting. **F** O-GlcN-mut relieved the ArgS-repressed de novo protein synthesis beyond HO-1 in BT-549 cells overexpressing either WT or O-GlcN-mut eIF2α. The cells were cultured under ± Arg for 24 h with the addition of AHA (250 μM) for the last 6 h, and protein input was visualized through Coomassie blue staining (*left panel*) and global de novo protein analysis was visualized using an anti-biotin antibody (*right panel*). **A**–**E** The data is presented as mean ± s.e.m. and was analyzed using either one-way ANOVA (for **A**–**C**) or two-way ANOVA followed by Tukey's multiple comparison test (for **D**, **E**). Statistical significance was determined with *p < 0.05; **p < 0.01; ***p < 0.001

Having established that eIF2α O-GlcNAcylation suppressed HO-1 expression in BT-549 cells subjected to ArgS above, we next wished to investigate whether the HO-1 translation is controlled by eIF2α O-GlcNAcylation. Along this line, spheres exhibited a decreasing HO-1 signal gradient from the peripheral region of spheres towards the core area even in the full medium (Fig. 5A). Indeed, the HO-1 signal reduction in BT-549 spheres was more noticeable in an -Arg medium compared to those in a complete medium (Fig. 5A). Altogether, these results suggested that the steady-state level of HO-1 is subjected to the regulation by a general lack of nutrients. To explore this further, immunohistochemical staining showed that HO-1 signals also deceased in xenografted tumors harvested from mice fed with -Arg diet compared with the mice fed with control diet (Additional file 1: Fig. S5A), providing support that Arg availability is a key regulator for HO-1 protein abundance. Intriguingly, while ArgS reduced steady-state HO-1 protein levels, ArgS induced *HMOX1* mRNA levels and the *HMOX1* antioxidant response element (ARE)-driven luciferase activities (Fig. 5B). Lastly, the treatment with proteasome inhibitor MG132 ([38], 10 μM) failed to rescue the HO-1 protein levels in the Arg-starved BT-549 cells despite its inhibitory effect on ATF4 induction in Arg-starved BT-549 cells (Fig. 5C). This observation argued that the proteasome-mediated protein degradation was not likely involved in HO-1 protein attenuation during ArgS. To reconcile the observed discordance between decreased HO-1 protein levels and increased *HMOX1* transcript levels in Arg-starved cells, we suspected that the translational suppression on *HMOX1* mRNA contributes to ArgS-promoted HO-1 protein reduction. To test this possibility, we utilized a Met analog, AHA, coupling with Click-iT reaction [47], to metabolically label and pull-down the nascent HO-1 in BT-549 cells overexpressing WT or O-GlcN-mut eIF2α under ± Arg conditions. Newly synthesized HO-1 protein levels were notably reduced upon ArgS in cells overexpressing WT eIF2α, compared to cells overexpressing O-GlcN-mut eIF2α (Fig. 5D, left panel, lanes 4, 6 versus lanes 3, 5). In contrast, O-GlcN-mut eIF2α unequivocally increased newly synthesized HO-1 protein in Arg-starved cells compared to WT eIF2α (Fig. 5D, left panel, lane 6 versus lane 4). This result indicated that the increased HO-1 protein levels in the Arg-starved, O-GlcN-mut eIF2α overexpressing cells resulted from the rescue of de novo HO-1 protein translation. However, we noticed that the steady-state level of ATF4 protein was significantly increased upon ArgS, de novo ATF4 level decreased after 24 h of ArgS (Fig. 5D, left panel, lane 4 versus lane 3). In a more detailed analysis of newly synthesized protein during the course of ArgS (6 h and 24 h), the de novo synthesized ATF4 level was higher at 6 h, then decreased at 24 h, post ArgS, indicating ArgS-induced ATF4 protein synthesis is an early-stage response to ArgS (Fig. 5E, lane 4 versus lane 6). Besides rescuing de novo HO-1 protein translation, O-GlcN-mut eIF2α also improved the translation of additional proteins with a wide range of molecular weights in the context of ArgS (Fig. 5F, *right panel*). Altogether, ArgS-induced eIF2α O-GlcNAcylation attenuates the translation of HO-1 and other proteins.

### eIF2α O-GlcNAcylation governs HO-1 expression independently of Ser51 phosphorylation

**Fig. 6:**
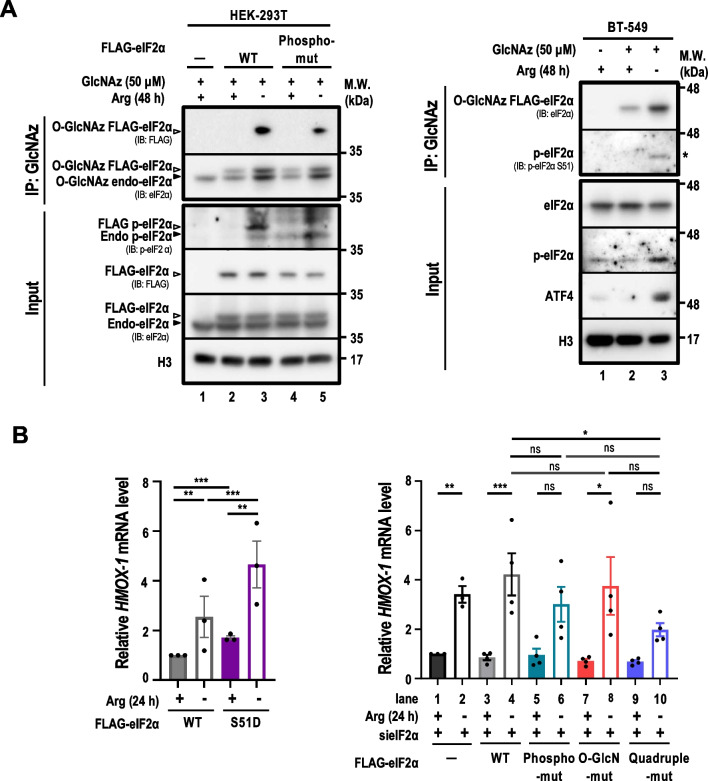
Co-existing eIF2α O-GlcNAcylation and phosphorylation regulate HO-1 expression in opposite manner. **A** O-GlcNAz-modified proteins in HEK293T cells overexpressing FLAG-tagged WT or phospho-mut eIF2α (*left panel*) and parental BT-549 cells (right panel) were pulled down and subjected to immunoblot analysis (n = 2). Negative controls were parental HEK293T cells without transient overexpression and BT-549 cells without GlcNAz labeling. HEK293T and BT-549 cells were treated with GlcNAz (50 μM, 48 h) in an -Arg medium prior to cell harvest. O-GlcNAz-modified proteins were collected and analyzed using an anti-FLAG or anti-eIF2α antibody to determine the FLAG-tagged exogenous or endogenous eIF2α O-GlcNAcylation levels. Black arrowheads indicate endogenous eIF2α, while white arrowheads indicate FLAG-tagged eIF2α. The asterisk (*) indicates phosphorylation of eIF2α on O-GlcNAz-modified eIF2α. **B** qRT-PCR analysis of *HMOX1* mRNA levels in parental BT-549 and eIF2α (WT or mutants) overexpressing cells subjected to ArgS for 24 h; n = 4. On the *left*, cells were transfected with FLAG-tagged WT or phospho-mimicking (S51D) eIF2α (2 μg) for 24 h prior to ArgS. Parental BT-549 cells serve as the baseline for HMOX1 expression. On the *right*, BT-549 cells stably overexpressing FLAG-tagged WT, phospho-mut, O-GlcN-mut, and quadruple-mut eIF2α were transfected with siRNA for eIF2α (30 nM) for 48 h prior to ArgS. *HMOX1* mRNA levels were determined by qRT-PCR and analyzed as described in Fig. 5C. Data are shown as mean ± s.e.m.; *p < 0.05; ***p < 0.001; Two-Way ANOVA followed by Tukey's multiple comparison test

Both eIF2α O-GlcNAcylation (Fig. 2A) and Ser51 phosphorylation (Fig. 4B, right panel, lane 2) were induced by ArgS. We also showed that ArgS was able to induce Ser51 phosphorylation on the O-GlcN-mut eIF2α (Additional file 1: Fig. S4D, left panel, lane 10). To test whether p-eIF2α affected eIF2α O-GlcNAcylation, immunoprecipitation coupled with Western analyses revealed that eIF2α O-GlcNAcylation signal was detected in the phospho-mut eIF2α, as in its counterpart FLAG-tagged WT, in Arg-starved HEK293T cells (Fig. 6A, left panel, lane 5 versus lane 3). Together with results shown in Fig. 4, Additional file 1: Fig. S4D, we suggest that eIF2α O-GlcNAcylation occurs irrespective of eIF2α Ser51 phosphorylation following ArgS. In addition, the pulled-down endogenous O-GlcNAcylated eIF2α was phosphorylated at Ser51 in Arg-starved BT-549 cells (Fig. 6A, right panel, lane 3). These results indicated that eIF2α O-GlcNAcylation and Ser51 phosphorylation coexist in the context of ArgS.

To further explore the role of eIF2α PTM in response to ArgS, a Ser51 phosphorylation-mimicking eIF2α was constructed by replacing Ser51 with an aspartate (Asp, D). However, despite increased *HMOX1* mRNA levels in full medium (Fig. 6B, left panel), the eIF2α S51D mutation, compared to WT, failed to affect the HO-1 protein levels in BT549 cells in full or -Arg medium (Additional file 1: Fig. S5B, top panel, lane 5, 6 versus lane 3, 4). Next, RNAs from BT-549 cells expressing either WT or various eIF2α mutants in the ± Arg were subjected to qRT-PCR analyses. As shown in Fig. 6B (*right panel*), only the quadruple-mut eIF2α showed a significant reduction in *HMOX1* mRNA levels compared to WT upon ArgS (Fig. 6B, right panel, lane 10 versus lane 4). Indeed, O-GlcN-mut eIF2α failed to reduce ArgS-mediated *HMOX1* transcriptional activation (Fig. 6B, right panel, lane 8 versus lane 4), confirming that the ArgS-decreased HO-1 protein level is not executed at the transcriptional level (Fig. 5C). Lastly, both O-GlcN-mut and quadruple-mut eIF2α significantly reduce CellROX signals under both ± Arg context (Additional file 1: Fig. S5C, lane 1 versus lane 3, 7 and lane 2 versus lane 4, 8), suggesting that eIF2α O-GlcNAcylation deficiency enables cells to have a better antioxidant capacity. Altogether, our results uncovered the previously unnoticed effect of eIF2α O-GlcNAcylation, independent of well-recognized Ser51 phosphorylation, suppresses HO-1 translation in the context of ArgS to promote ROS-mediated cell death.

## Discussion

In this study, we identify a key role for eIF2α O-GlcNAcylation in the adaptive response to the stress rejiggered by the depletion of extracellular Arg. We show that eIF2α O-GlcNAcylation is essential for the suppression of de novo HO-1 protein synthesis, which serves a key protective role against ROS upon Arg removal. Translational control is frequently used in response to various stress stimuli to provide an immediate and selective change in protein levels. In this regard, p-eIF2α has long been recognized as a defining step in stress-regulated translational initiation [8]. Herein, we report that ArgS-induced eIF2α O-GlcNAcylation decreases the expression of the antioxidant protein HO-1 via attenuation of its translation, resulting in an accumulation of intracellular ROS levels (Additional file 1: Fig. S6).

Translation initiation is the rate-limiting step in the regulation of translation [8]. Under stress, the p-eIF2α effectively reduces the level of active eIF2, thus inhibiting mRNA translation initiation [12] and global protein synthesis [1]. However, the translation of ATF4 under stress is selectively enhanced by p-eIF2α [58]. Because that eIF2α Ser51 phosphorylation inhibits eIF2B and delays GTP reloading in the ternary complex [58], the reduced level of eIF2α-preinitiation complex will then scan through the inhibitory upstream open reading frame 2 (uORF2) and selectively initiate the translation of stress-induced proteins, such as ATF4 [8]. Along this line, there is a stop codon, UAG, in the uORF located at the 5’-UTR of *HMOX1* mRNA [59]. We speculate that O-GlcNAcylation of eIF2α might alter the structure of preinitiation complex to favor the scanning of uORF in the context of AS. The ribosome is then either released or stalled in the uORF due to the stop codon to prevent the translation of the main ORF, resulting in translational repression of HO-1. Our results imply that Arg-starved cells utilize eIF2α O-GlcNAcylation to suppress the translation of HO-1, a protein that is critical for surviving oxidative stress under ArgS (Additional file 1: Fig. S6).

Our studies on Arg deprivation have shown that a shortage of Arg negatively impacts mitochondrial function, resulting in decreased levels of α-ketoglutarate (α-KG) and changes in epigenetic regulation [7, 33, 35, 36]. These studies revealed that α-KG acts as a cofactor for histone demethylases, and its decrease results in an increase in the number of repressive marks on genes related to mitochondrial functions such as oxidative phosphorylation and the synthesis of purines and pyrimidines. We propose that this is due to the accumulation of ROS caused by disrupted mitochondria during Arg deficiency, leading to heightened expression of MYC [60] and MYC-regulated O-GlcNAcylation [61]. Indeed, O-GlcNAcylation is a post-translational modification that impacts the activity and stability of enzymes and proteins involved in cellular protection against oxidative stress. For instance, O-GlcNAcylation has been shown to regulate the activity and stability of the tumor suppressor protein p53 [62], which can help protect cells from oxidative damage by triggering cell cycle arrest or apoptosis in response to DNA damage. Another example is KEAP1, a protein that activates the expression of antioxidant and detoxifying enzymes such as glutathione S-transferase and NAD(P)H:quinone oxidoreductase 1. Moreover, O-GlcNAcylation has been shown to positively regulate the NRF2 signaling pathway, which helps cells combat oxidative stress [63]. However, the specific effects of O-GlcNAcylation on these proteins can vary depending on the type of cell, tissue, and oxidative stress conditions. In our study, we demonstrated that O-GlcNAcylation of eIF2α results in an increase in ROS through the inhibition of antioxidant HO-1 translation during ArgS. In conclusion, the relationship between O-GlcNAcylation and oxidative stress is complex and more research is required to fully understand its role in protecting (or not) cells from oxidative stress.

It is noteworthy that our GlcNAz-based O-GlcNAc proteomic analysis identified 13 O‑GlcNAcylated proteins that partake in eukaryotic protein translation (Fig. 1D). Furthermore, literature has reported that the O-GlcNAcylation of eIF3a, eIF4A, and eIF4G altered the complex assembly that promotes translation or modulates CAP-dependent translational initiation [64–66]. Amino acid shortage-induced de-O-GlcNAcylation of eIF3a destabilizes the association between eIF3 and 43S preinitiation complex, leading to the recycling of eIF3 from the elongating 80S ribosomes [64]. In the eIF4F complex, O-GlcNAcylation plays an opposing role in regulating the functions of eIF4A and eIF4G subunits in translation [65]. O-GlcNAcylation of eIF4A disrupts the assembly of the eIF4F complex by interfering with its interaction with eIF4G, resulting in translational inhibition [65, 66]. In contrast, eIF4G O-GlcNAcylation promotes the interaction of eIF4G with poly (A)-binding protein and poly (A) RNA, which is an essential step in supporting translational initiation [65, 66]. Moreover, eIF4A and eIF4G O-GlcNAcylation are cell context- or cell cycle-dependent, indicating that the O-GlcNAcylation of eIF4F complex is mechanistically linked to the protein synthesis fine-tuning [65]. However, we did not detect any O-GlcNAcylation level alteration of previously identified eIF3a, eIF4A and eIF4G in the context of ArgS (Fig. 1D), supporting the diverse regulation of translation by O-GlcNAcylation.

Furthermore, our study offers a deeper understanding of how the antioxidant protein HO-1 is suppressed, particularly in the context of ArgS, through eIF2α O-GlcNAcylation. Our study raises several questions for future research. First, it is known that during Arg limitation, both eIF2α O-GlcNAcylation and ribosome pausing contribute to translational suppression [67, 68]. However, it is not clear how increased eIF2α and eIF4A O-GlcNAcylation or decreased eIF2γ, eIF4G1, and EF1α O-GlcNAcylation (Fig. 1D) may contribute to ribosome pausing specifically in the context of ArgS. It is currently unclear whether eIF2α O-GlcNAcylation is involved in regulating other stress responses induced by limitations in Arg availability. Therefore, identifying the specific mRNAs whose translation is inhibited by O-GlcNAcylated eIF2α during ArgS would be valuable. Moreover, the O-GlcNAcylation landscape changes significantly in response to various metabolic stressors, which may have distinct effects on the O-GlcNAcylation of translational initiators like eIF4G1, eIF4A1, and eIF2α. This suggests that different metabolic stressors may differentially impact the enzymatic activity or substrate specificity of OGT or OGA. Another related question is how a single OGT or OGA enzyme recognizes and site-specifically modifies a plethora of substrates under a variety of metabolic stress. Several mechanisms have been proposed for OGT and OGA to recognize their specific substrates [69–72], but it is still unclear how ArgS regulates OGT and OGA differentially with respect to the diverse set of translational factors and regulators in protein synthesis. It remains unknown whether other PTMs, such as ubiquitination and phosphorylation, play a role in regulating ArgS-induced OGT or OGA activity. Perhaps eIF2α O-GlcNAcylation is dispensable when nutrients are available, as in most healthy tissues. However, in tumors where cells are proliferating and nutrients are scarce, eIF2α O-GlcNAcylation may become crucial in regulating de novo protein synthesis.

In summary, our findings demonstrate a crucial role for eIF2α O-GlcNAcylation in inhibiting de novo HO-1 synthesis in response to extracellular Arg restriction. Further investigation is necessary to uncover how translation machinery is specifically regulated under various (patho)physiological conditions, such as diabetes, neurodegenerative diseases, or autoimmune diseases. Additionally, it would be important to explore the mechanisms through which eIF2α O-GlcNAcylation regulates tumor growth and its potential role in modulating therapeutic responses.

## Conclusions

Arg, a non-essential amino acid, is an important component of protein synthesis and plays a role in regulating various metabolic pathways, including protein O-GlcNAcylation. ArgS is an established therapeutic approach. This study is the first to demonstrate that Arg availability modulates eIF2α O-GlcNAcylation, which in turn impacts the production of the antioxidant protein HO-1 and the associated antioxidant process.

## Supplementary Information

**Additional file 1: Figure S1.** The ArgS treatment leads to a reduction in global O-GlcNAcylation levels and affects the transcription of HBP and O-GlcNAc recycling enzyme genes.Immunoblot analysis of OGA and OGT in the whole cell extracts from BT-549 cells cultured in the -Gln or -Glc m4dium for 24 h. One representative immunoblotis shown. The relative OGA and OGT protein levels are determined after normalizing against the densitometric signal intensity of the OGA or OGT in the control group, which was set as 1 after normalization with actin or H3 signal.A schematic overview of HBP. The HBP enzymesand metabolitesare depicted.Gene expression of the indicated genes in BT-549 cells after incubation in full medium for 48 h or -Arg medium for 24 and 48 h, respectively. The results of the qRT-PCR analysis of the mRNA encoding HBP enzymes shown in the left panel in BT-549 cells after ArgS treatment for 24 and 48 h, respectively. The qRT-PCR analyses were performed using gene-specific primer pairs, and the 2^–∆∆Ct^ method was used to analyze the results. ΔΔCT = ΔCT− ΔCT.The Staudinger ligation was performed in protein lysates where O-GlcNAz-modified proteins were conjugated with phosphine-PEG3-biotin. The O-GlcNAz-modified proteins were then pulled-down using streptavidin.A heat map shows the abundance ratioof 2054 quantified O-GlcNAz-modified proteins. The scale of the heat map is limited to; n=4.GlcNAz-labeled proteins in BT-549 cells were pulled down and subjected to immunoblot analyses. BT-549 cells were maintained in -Gln and -Glc medium supplemented with GlcNAzfor 48 h prior to cell harvest. O-GlcNaz-modified proteins were collected, followed by immunoblot analysis using an anti-eIF2α antibody to determine the endogenous eIF2α O-GlcNAcylation levels.Data are shown as mean ± s.e.m.; *: *p*<0.05; **: *p*<0.01; ***: *p*<0.001; One-Way ANOVA. **Figure S2.** Antioxidant gene expression analyses in breast carcinoma samples.Immunoblots of HO-1 in BT-549 and MDA-MB-2321 cells subjected to ArgS or ADI-PEG20treatment for 24 and 48 h; n=3.Immunoblot analysis of HO-1 in BT-549 cells subjected to ArgS for 24 h, followed by Arg recovery for 6, 12, and 24 h. One representative immunoblotis shown.Antioxidant gene *HMOX1, CISD2*, *SOD1*, and *GSR *expression in the normal, tumor and metastatic tissues collected from breast cancer patients were examined.Pancancer analysis of *HMOX1* expression across normaland tumor tissues. 17 cancer types in TCGA Pan-Cancer study having >5 normal samples were presented as indicated. Standard boxplots were applied to visualize the log2-transformed *HMOX1* expression levelsand the number of samples was labeled at the bottom.*HMOX1 *expressionin breast tumors across different molecular subtypes and adjacent normal tissues. The number of samples was labeled at the bottom.*: *p*<0.05; **: *p*<0.01; ***: *p*<0.001; N.S.: *p*>0.05; Wilcoxon tests; *p*-values were adjusted for multiple comparison using Bonferroni method with the normal samples set as the reference group.Kaplan-Meier survival curves comparing the HO-1 protein levels and overall survivalrates of breast cancer patients. 58 breast tumor samples expressing detectable/no detectable HO-1 in the Tang database were applied to the comparison using log-rank test. **Figure S3.** HO-1 expression enhances cell recovery from ArgS.Immunoblot analysis of HO1 in different breast cancer cell lines. The whole cell lysates were collected from the different cell lines grown in complete medium.Immunoblot analysis of HO-1 in BT-549 cellstransfected with *siHMOX1*or treated with ZnPPIX, and in MDA-MB-231 cellsstably overexpressing HO-1 or treated with COPPIX.Measurement of ROS levels in BT-549 and MDA-MB-231 cells subjected to ArgS for 48 h. ROS levels were assessed using DCF-DA oxidation; n=3.Comparison of relative cell viability in BT-549 and MDA-MB-231 cells subjected to ArgS for 24 and 48 h. Assessment of relative cell recovery of MDA-MB-231 cells after treatment with ADI-PEG20for 24 h. The ADIPEG20 treatment was terminated after 24 h; n=3. Comparison of cell recovery in MDA-MB-231 cells overexpressing HO-1 after 24-h treatment with ADI-PEG20; n=3. The ADI-PEG20 treatmentwas terminated by replacing the medium with complete medium. Cell recovery was monitored daily from day 0 to day 3using ACP assays.Assessment of cell migration of MDA-MB-231 cells subjected to ArgS for 24 h; n=3. Cell migration was determined as the percentage of confluence within the gap area.Data are shown as mean ± s.e.m.; *: *p*<0.05; **: *p*<0.001; ***: *p*<0.001; determined by one-way ANOVAor Two-Way ANOVA followed by Tukey's multiple comparison test. **Figure S4.** eIF2α S51 phosphorylation does not decrease HO-1 expression during ArgS stress.Immunoblot analysis of HO-1 protein abundance in BT-549 cells with defective eIF2α phosphorylation. The effect of defective eIF2α phosphorylation on HO-1 expression was analyzed through the following treatments: 24 h ArgS with tauroursodeoxycholic acid, ISRIB, and phospho-mutant eIF2α. Furthermore, the expression of HO-1 in BT-549 cells stably overexpressing wild-type or O-GlcN-mut or phosphomut eIF2α was analyzed after 24 h of ArgS treatment. One representative immunoblotis shown, with H3 serving as a loading control. The results are shown with parental BT-549 cells serving as a negative control for eIF2α stable expression. The cells were transfected with *si-eIF2α*or* siCtrl*for 48 h prior to ArgS treatment. The relative level of HO-1 proteinwas determined by comparing the densitometric tracing of the HO-1 signal in each experimental condition to the reference HO-1 signal, after normalization with H3. A value greater than 1 indicates an increase in abundance relative to the control, while a value less than 1 indicates a decrease in abundance. Data are presented as mean ± s.e.m.; *: *p*<0.05; **: *p*<0.01; ***: *p*<0.001; Two-Way ANOVA followed by Tukey’s multiple comparison test. **Figure S5.** HO-1 IHC staining in BT-549 xenografted tumor.Representative IHC staining shows the levels of HO-1 protein in two xenografted tumors harvested from mice that were fed a control dietand -Arg diet.Immunoblot analysis of HO-1 expression in BT-549 cells that were overexpressing a phosphomimickingform of eIF2α. Cells were transfected with FLAG-tagged wild-type or S51D eIF2α expression constructs and then subjected to ArgS. The black arrowheads indicate endogenous eIF2α, and the white arrowheads indicate FLAG-tagged eIF2α. The relative level of HO-1 protein was determined by comparing the densitometric HO-1 signal in the experimental conditions to the reference HO-1. The values of reference HO-1 were set to 1 after normalization with H3, which was used as a loading control.ROS levels in BT-549 cells that were stably expressing wild-type, phosphomut, O-GlcN-mut, or quadruple-mut eIF2α after being subjected to ArgS for 48 h. The ROS level was quantified with CellROX™ via flow cytometry, and the relative oxidized CellROX level is shown after normalization with the value of the control, which was set to 1. The data are shown as mean ± s.e.m.; ns: non-significant, *: *p*<0.05; **: *p*<0.01; ***: *p*<0.001. The analysis was performed using a Two-Way ANOVA followed by Tukey's multiple comparison test. **Figure S6.** Overall summary. Our data suggest that O-GlcNAcylation of eIF2α is the primary mechanism for downregulating HO-1 protein translation during ArgS. The downregulation of HO- 1 protein leads to an increase in ROS levels and a reduction in cell recovery from ArgS and migratory ability. ArgS induces O-GlcNAcylation on eIF2α, which is considered a stress response to the treatment. The inhibitory effect of increased eIF2α O-GlcNAcylation on antioxidant protein translation reveals a novel mechanism by which ArgS may serve as a potential anti-cancer treatment. 

**Additional file 2: Table S1.** Primer sequences for qRT-PCR. **Table S2.** Antibodies. **Table S3. **Primer sequences for mutagenesisof eIF2α.

**Additional file 3:** **Table S4.** Proteomic raw data. **Table S5.** AS pathway

## Data Availability

The O-GlcNAcylation proteomic datasets generated and/or analyzed during the current study are available in the Proteome X Change repository (the access number (pending) will be provided once it is available).

## Abbreviations

Arg: Arginine-free
Glc: Glucose starvation
Gln: Glutamine starvation
Ac4GlcNAz: Tetraacetylated N-azidoacetylglucosamine
ACP: Acid phosphatase
ADI-PEG20: Arginine deiminase pegylated with 20,000-molecular-weight polyethylene glycol
AHA: L-azidohomoalanine
Ala, A: Alanine
ARE: Antioxidant response element
Arg: Arginine
ArgS: Arginine shortage, arginine starvation
Asp, D: Aspartate
ATCC: American type culture collection
ATF4: Activating transcription factor 4
BSA: Bovine serum albumin
CISD2: CDGSH iron-sulfur domain-containing protein 2
CoPPIX: Cobalt protoporphyrin
Ctrl: Control
DCF-DA: 2′-7′-Dichlorodihydrofluorescein diacetate
ddH_2_O: Double distilled water
DMEM: Dulbecco’s modified eagle's medium
DTT: Dithiothreitol
eIF: Eukaryotic translation initiation factor
EIF2S1: Eukaryotic translation initiation factor 2 subunit alpha (eIF2α)
ER: Endoplasmic reticulum
FBS: Fetal bovine serum
FDR: False discovery rate
GFP: Green fluorescent protein
Glc: Glucose
GlcNAz: N-azidoacetylglucosamine tetraacylated
Gln: Glutamine
GSR: Glutathione reductase
HBP: Hexosamine biosynthesis pathway
HEK293T: Human embryonic kidney 293 cells containing SV40 T-antigen
HO-1, *HMOX1*: Heme oxygenase-1
IHC: Immunohistochemistry
ISR: Integrated stress response
ISRIB: Integrated stress response inhibitor
α-KG: α-Ketoglutarate
LFQ: Label-free quantification
Met: Methionine
NSG: NOD.Cg-prkdcscidil2rgtm1wjl/szj
O.C.T.: Tissue-tek^®^ optimal cutting temperature
OE: Overexpressing
OGA: O-GlcNAcase
O-GlcN-mut: O-GlcNAcylation-defective S219A/T239A/T241A-eIF2α
O-GlcNAc: O-linked N-acetylglucosamine
O-GlcNAcylation: O-linked β-N-acetylglucosaminylation
OGT: O-GlcNAc transferase
p-eIF2α: Ser51-phosporylated eIF2α or phospho-eIF2α
PBS: Phosphate-buffered saline
Phospho-mut: Phosphorylation-defective S51A-eIF2α
PEG: Polyethylene glycol
pNPP: 4-Nitrophenyl phosphate disodium salt hexahydrate
PTM: Post-translational modification
ROS: Reactive oxygen species
RSEM: RNA-Seq by Expectation–Maximization
SDS: Sodium dodecyl-sulfate
S.E.M.: Standard error of the mean
SOD-1: Superoxide dismutase type 1
TCGA: The cancer genome atlas
TEAB: Tetraethylammonium bromide
Thr, T: Threonine
TN: Tunicamycin
TUDCA: Tauroursodeoxycholic acid
UDP-GlcNAc: Uridine diphosphate N-acetylglucosamine
UPR: Unfolded protein response
uORF: Upstream open reading frame 2
WT: Wild type
UTR: Untranslated region
ZnPPIX: Zinc protoporphyrin-9
