# Supplementary material for: Extracellular arginine availability modulates eIF2α O-GlcNAcylation and heme oxygenase 1 translation for cellular homeostasis
Source: J Biomed Sci. 2023 May 22;30:32. doi: 10.1186/s12929-023-00924-4 (PMC10201738; doi:10.1186/s12929-023-00924-4)
Supplement: Supplementary file 1 — Additional file 1: Figure S1. The ArgS treatment leads to a reduction in global O-GlcNAcylation levels and affects the transcription of HBP and O-GlcNAc recycling enzyme genes.Immunoblot analysis of OGA and OGT in the whole cell extracts from BT-549 cells cultured in the -Gln or -Glc m4dium for 24 h. One representative immunoblotis shown. The relative OGA and OGT protein levels are determined after normalizing against the densitometric signal intensity of the OGA or OGT in the control group, which was set as 1 after normalization with actin or H3 signal.A schematic overview of HBP. The HBP enzymesand metabolitesare depicted.Gene expression of the indicated genes in BT-549 cells after incubation in full medium for 48 h or -Arg medium for 24 and 48 h, respectively. The results of the qRT-PCR analysis of the mRNA encoding HBP enzymes shown in the left panel in BT-549 cells after ArgS treatment for 24 and 48 h, respectively. The qRT-PCR analyses were performed using gene-specific primer pairs, and the 2–∆∆Ct method was used to analyze the results. ΔΔCT = ΔCT− ΔCT.The Staudinger ligation was performed in protein lysates where O-GlcNAz-modified proteins were conjugated with phosphine-PEG3-biotin. The O-GlcNAz-modified proteins were then pulled-down using streptavidin.A heat map shows the abundance ratioof 2054 quantified O-GlcNAz-modified proteins. The scale of the heat map is limited to; n=4.GlcNAz-labeled proteins in BT-549 cells were pulled down and subjected to immunoblot analyses. BT-549 cells were maintained in -Gln and -Glc medium supplemented with GlcNAzfor 48 h prior to cell harvest. O-GlcNaz-modified proteins were collected, followed by immunoblot analysis using an anti-eIF2α antibody to determine the endogenous eIF2α O-GlcNAcylation levels.Data are shown as mean ± s.e.m.; *: p<0.05; **: p<0.01; ***: p<0.001; One-Way ANOVA. Figure S2. Antioxidant gene expression analyses in breast carcinoma samples.Immunoblots of HO-1 in BT-549 and MDA-MB-2321 cells subjected to [file 12929_2023_924_MOESM1_ESM.pdf]

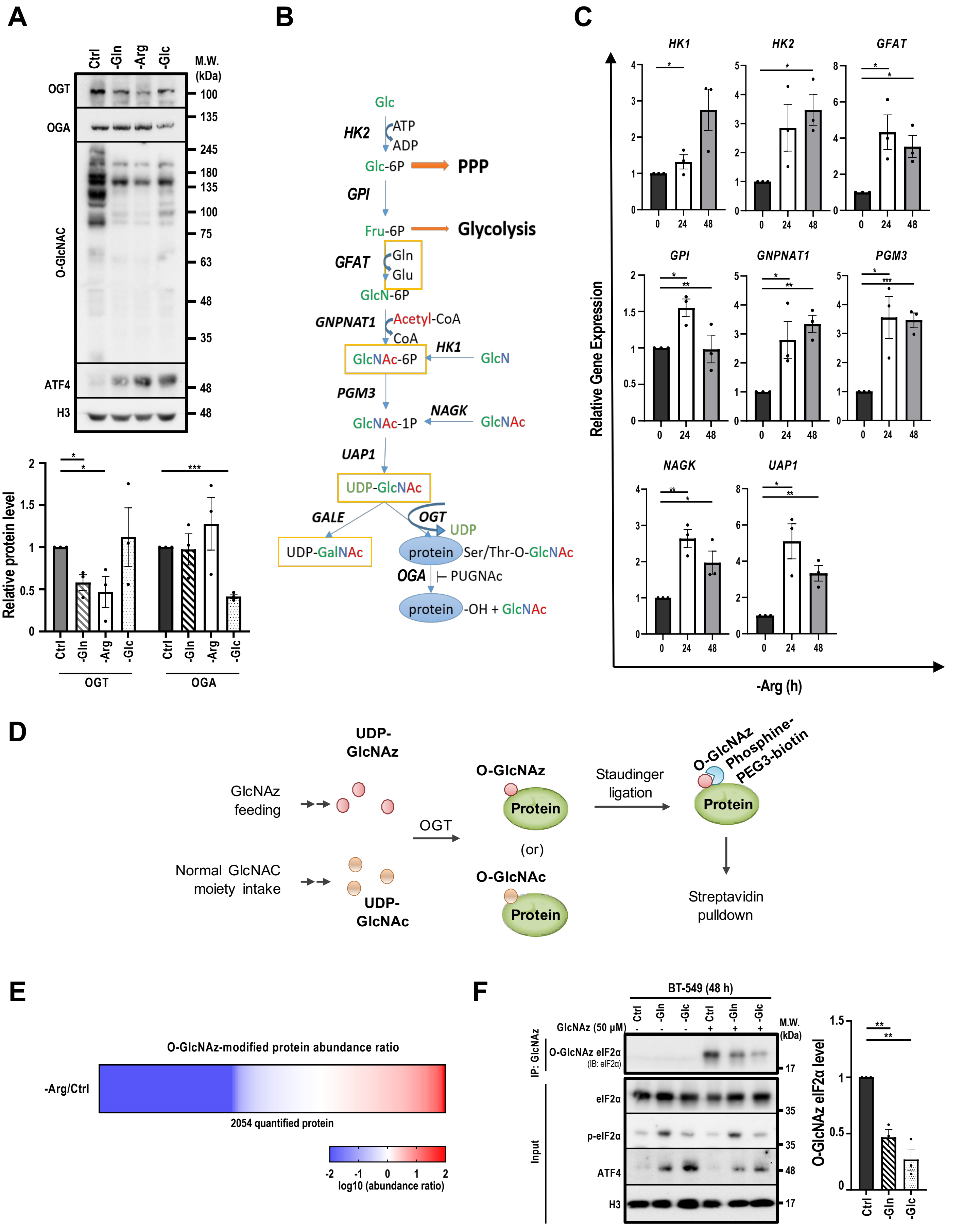

Supplemental Fig. S1

Fig. S1. The ArgS treatment leads to a reduction in global O-GlcNAcylation levels and affects the transcription of HBP and O-GlcNAc recycling enzyme genes. (**A**) Immunoblot analysis of OGA and OGT in the whole cell extracts from BT-549 cells cultured in the -Gln or -Glc m4dium for 24 h. One representative immunoblot (*left panel*, n=3) is shown. The relative OGA and OGT protein levels are determined after normalizing against the densitometric signal intensity of the OGA or OGT in the control group (Ctrl), which was set as 1 after normalization with actin or H3 signal (serving as a loading control). (**B**) A schematic overview of HBP. The HBP enzymes (*italicized*) and metabolites (GlcNAc moiety) are depicted. (**C**) Gene expression of the indicated genes in BT-549 cells after incubation in full medium for 48 h or -Arg medium for 24 and 48 h, respectively. The results of the qRT-PCR analysis of the mRNA encoding HBP enzymes shown in the left panel in BT-549 cells after ArgS treatment for 24 and 48 h, respectively. The qRT-PCR analyses were performed using gene-specific primer pairs, and the $2^{-\Delta\Delta Ct}$ method was used to analyze the results. ΔΔCT = ΔCT (a target sample) − ΔCT (a reference sample). (**D**)The Staudinger ligation was performed in protein lysates where O-GlcNAz-modified proteins were conjugated with phosphine-PEG3-biotin. The O-GlcNAz-modified proteins were then pulled-down using streptavidin (*left panel*). (**E**) A heat map shows the abundance ratio (-Arg/Ctrl) of 2054 quantified O-GlcNAz-modified proteins. The scale of the heat map is limited to (-2, 2); n=4. (**F**) GlcNAz-labeled proteins in BT-549 cells were pulled down and subjected to immunoblot analyses (n=3). BT-549 cells were maintained in -Gln and -Glc medium supplemented with GlcNAz (50 μM) for 48 h prior to cell harvest. O-GlcNaz-modified proteins were collected, followed by immunoblot analysis using an anti-eIF2α antibody to determine the endogenous eIF2α O-GlcNAcylation levels. (**A**, **C**, **F**) Data are shown as mean ± s.e.m.; *: p<0.05; **: p<0.01; ***: p<0.001; One-Way ANOVA.

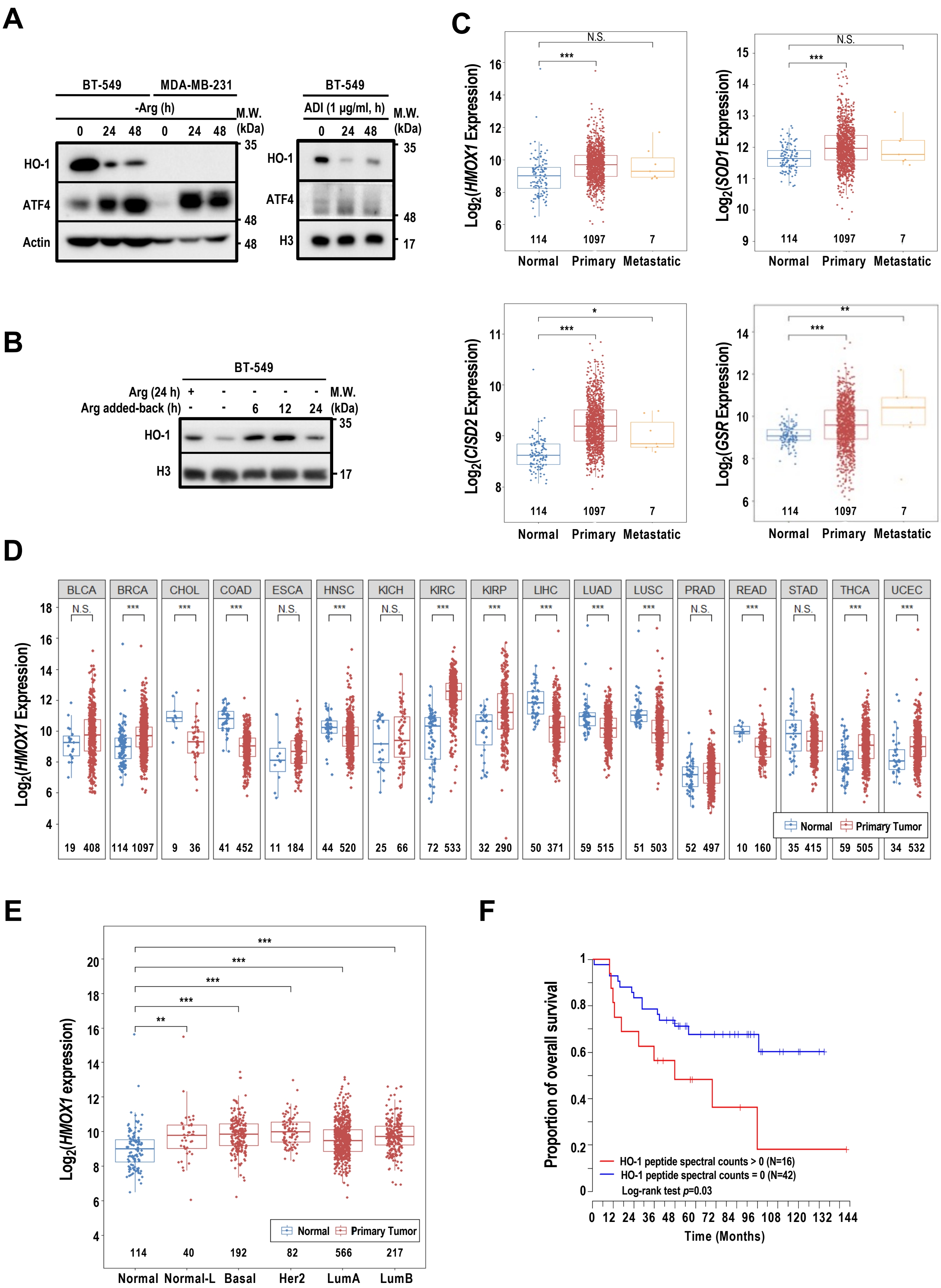

Supplemental Fig. S2

Fig. S2. Antioxidant gene expression analyses in breast carcinoma samples. (**A**) Immunoblots of HO-1 in BT-549 and MDA-MB-2321 cells subjected to ArgS or ADI-PEG20 (1 μg/ml) treatment for 24 and 48 h; n=3. (**B**) Immunoblot analysis of HO-1 in BT-549 cells subjected to ArgS for 24 h, followed by Arg recovery for 6, 12, and 24 h. One representative immunoblot (n=2) is shown. (**C**) Antioxidant gene *HMOX1, CISD2*, *SOD1*, and *GSR* expression in the normal, tumor and metastatic tissues collected from breast cancer patients were examined (Normal n=114, Primary n=1097, and metastatic tissue n=7) (RNA-Seq data; TCGA database). (**D**) Pan-cancer analysis of *HMOX1* expression across normal (blue) and tumor tissues (red). 17 cancer types in TCGA Pan-Cancer study having >5 normal samples were presented as indicated. Standard boxplots were applied to visualize the log2-transformed *HMOX1* expression levels (RSEM) and the number of samples was labeled at the bottom. (**E**) *HMOX1* expression (RNA-Seq data; TCGA) in breast tumors across different molecular subtypes and adjacent normal tissues. The number of samples was labeled at the bottom. (**C, D, E**) *: $p<0.05$; **: $p<0.01$; ***: $p<0.001$; N.S.: $p>0.05$; Wilcoxon tests; *p*-values were adjusted for multiple comparison using Bonferroni method with the normal samples set as the reference group. (**F**) Kaplan-Meier survival curves comparing the HO-1 protein levels and overall survival (OS) rates of breast cancer patients. 58 breast tumor samples expressing detectable/no detectable HO-1 in the Tang database were applied to the comparison using log-rank test.

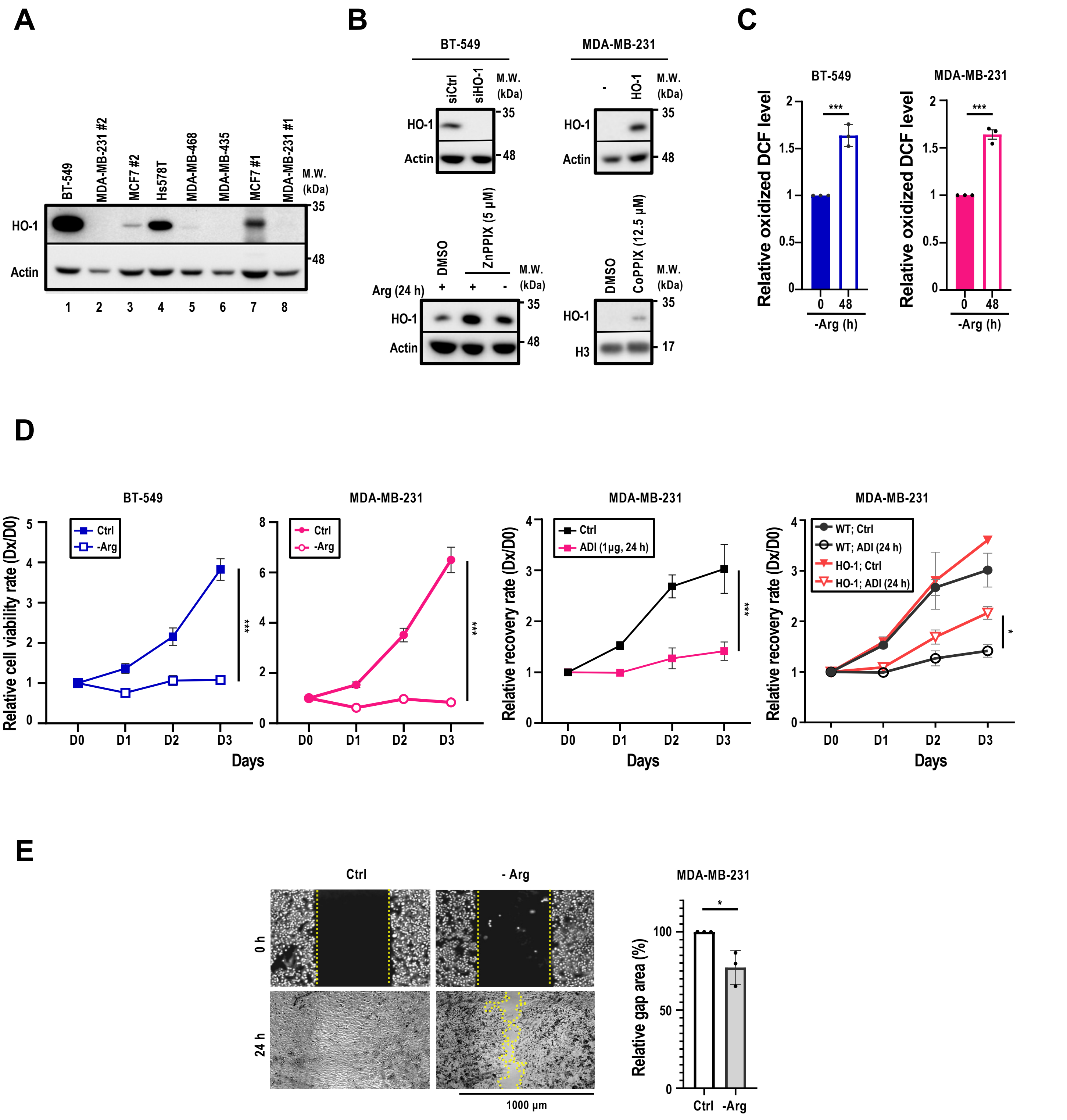

Supplemental Fig. S3

Fig. S3. HO-1 expression enhances cell recovery from ArgS. (**A**) Immunoblot analysis of HO-1 in different breast cancer cell lines. The whole cell lysates were collected from the different cell lines grown in complete medium. (**B**) Immunoblot analysis of HO-1 in BT-549 cells (*top panel*) transfected with *siHMOX1* (30 nM) or treated with ZnPPIX (5 μM), and in MDA-MB-231 cells (*bottom panel*) stably overexpressing HO-1 or treated with COPPIX (12.5 μM). (**C**) Measurement of ROS levels in BT-549 and MDA-MB-231 cells subjected to ArgS for 48 h. ROS levels were assessed using DCF-DA oxidation; n=3. (**D**) Comparison of relative cell viability in BT-549 and MDA-MB-231 cells subjected to ArgS for 24 and 48 h (*left two panels*). Assessment of relative cell recovery of MDA-MB-231 cells after treatment with ADI-PEG20 (1 μg/ml) for 24 h. The ADI-PEG20 treatment was terminated after 24 h; n=3 (*3rd panel from the left*). Comparison of cell recovery in MDA-MB-231 cells overexpressing HO-1 after 24-h treatment with ADI-PEG20 (1 μg/ml); n=3 (*right panel*). The ADI-PEG20 treatment (24 h) was terminated by replacing the medium with complete medium. Cell recovery was monitored daily from day 0 to day 3 (D0-D3, as indicated) using ACP assays. (**E**) Assessment of cell migration of MDA-MB-231 cells subjected to ArgS for 24 h; n=3. Cell migration was determined as the percentage of confluence within the gap area. (**C**-**E**) Data are shown as mean ± s.e.m.; *: p<0.05; **: p<0.001; ***: p<0.001; determined by one-way ANOVA (**C**, **D**) or Two-Way ANOVA followed by Tukey's multiple comparison test (**E**).

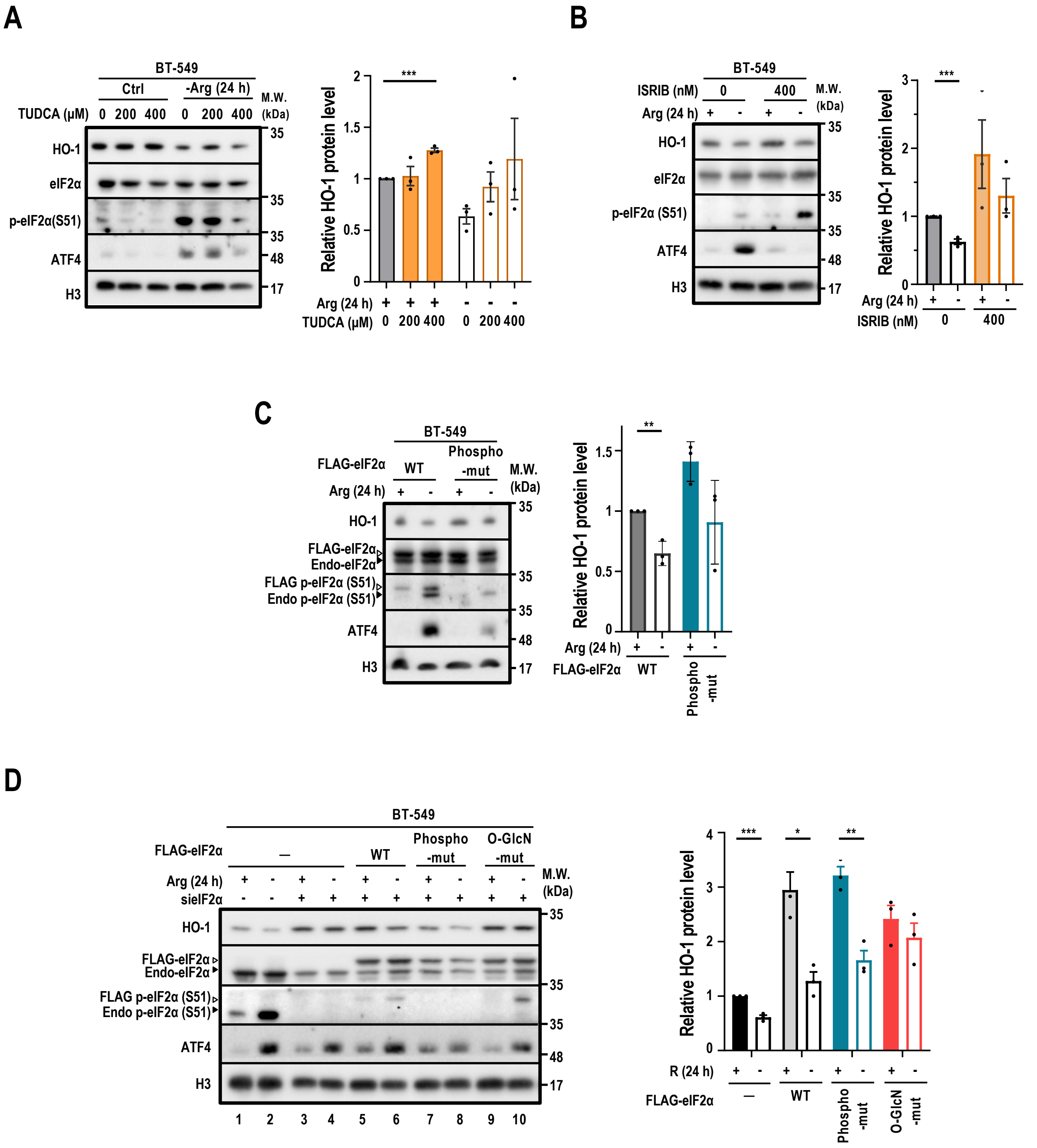

Supplemental Fig. S4

Fig. S4. eIF2α S51 phosphorylation does not decrease HO-1 expression during ArgS stress. (**A-D**) Immunoblot analysis of HO-1 protein abundance in BT-549 cells with defective eIF2α phosphorylation. The effect of defective eIF2α phosphorylation on HO-1 expression was analyzed through the following treatments: 24 h ArgS with tauroursodeoxycholic acid (TUDCA; 200 and 400 μM) (**A**), ISRIB (400 nM) (**B**), and phospho-mutant eIF2α (**C**). Furthermore, the expression of HO-1 in BT-549 cells stably overexpressing wild-type or O-GlcN-mut or phospho-mut eIF2α was analyzed after 24 h of ArgS treatment (**D**). One representative immunoblot (n=3) is shown, with H3 serving as a loading control. The results are shown with parental BT-549 cells serving as a negative control for eIF2α stable expression. The cells were transfected with *si-eIF2α* (3-UTR) or *siCtrl* (30 nM) for 48 h prior to ArgS treatment. The relative level of HO-1 protein (*right panels*) was determined by comparing the densitometric tracing of the HO-1 signal in each experimental condition to the reference HO-1 signal (Ctrl; no treatment), after normalization with H3 (as a loading control). A value greater than 1 indicates an increase in abundance relative to the control, while a value less than 1 indicates a decrease in abundance. Data are presented as mean ± s.e.m.; *: $p<0.05$; **: $p<0.01$; ***: $p<0.001$; Two-Way ANOVA followed by Tukey's multiple comparison test.

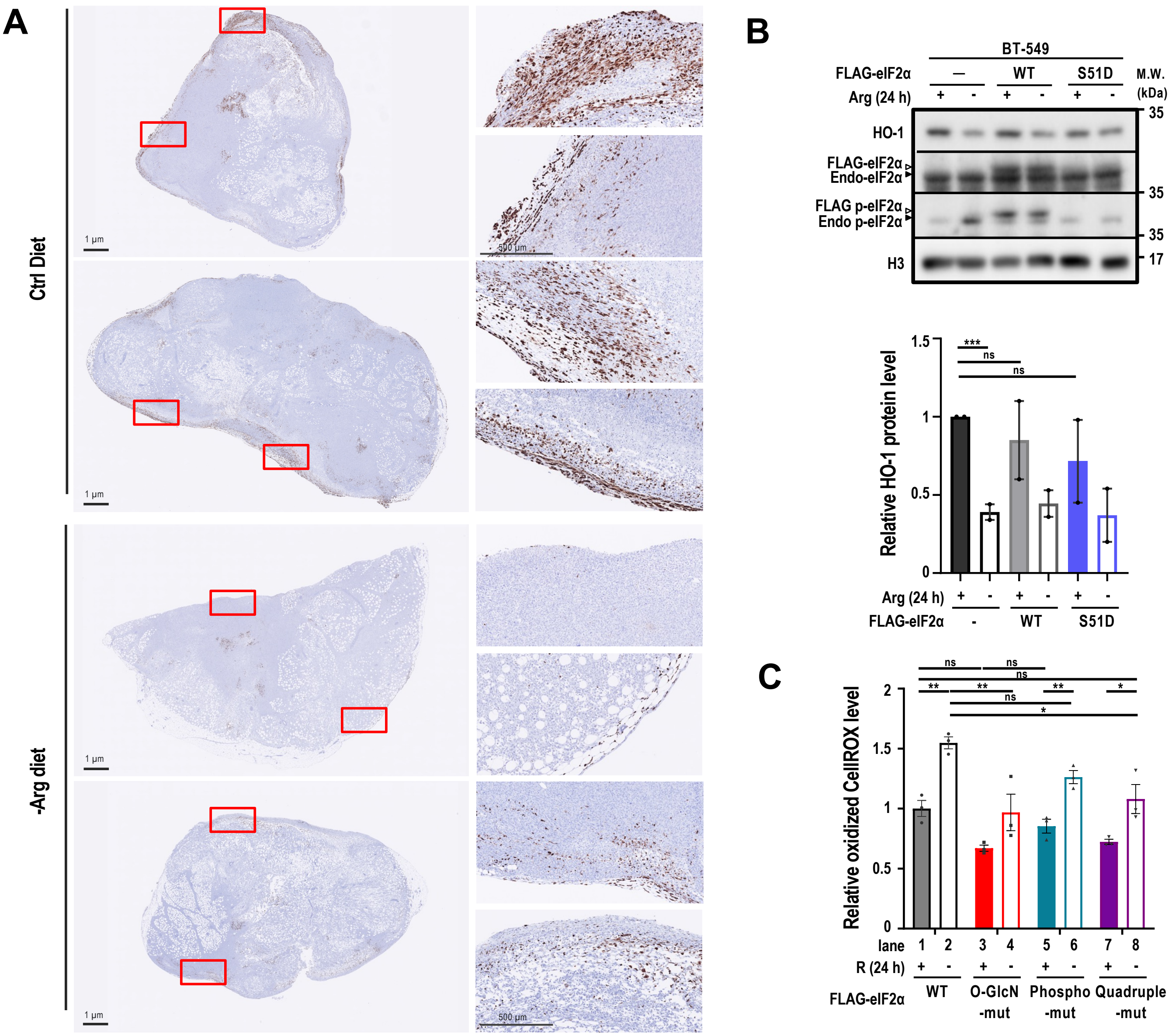

Supplemental Fig. S5

Fig. S5. HO-1 IHC staining in BT-549 xenografted tumor. (**A**) Representative IHC staining shows the levels of HO-1 protein in two xenografted tumors harvested from mice that were fed a control diet (*upper panels*) and -Arg diet (*lower panels*). (**B**) Immunoblot analysis of HO-1 expression in BT-549 cells that were overexpressing a phospho-mimicking (S51D) form of eIF2α. Cells were transfected with FLAG-tagged wild-type or S51D eIF2α expression constructs and then subjected to ArgS. The black arrowheads indicate endogenous eIF2α, and the white arrowheads indicate FLAG-tagged eIF2α. The relative level of HO-1 protein was determined by comparing the densitometric HO-1 signal in the experimental conditions to the reference HO-1 (eIF2α wild-type; +Arg). The values of reference HO-1 were set to 1 after normalization with H3, which was used as a loading control. (**C**) ROS levels in BT-549 cells that were stably expressing wild-type, phospho-mut, O-GlcN-mut, or quadruple-mut eIF2α after being subjected to ArgS for 48 h. The ROS level was quantified with CellROX™ via flow cytometry, and the relative oxidized CellROX level is shown after normalization with the value of the control (eIF2α WT, +Arg), which was set to 1. The data are shown as mean ± s.e.m.; ns: non-significant, *: $p<0.05$; **: $p<0.01$; ***: $p<0.001$. The analysis was performed using a Two-Way ANOVA followed by Tukey's multiple comparison test.

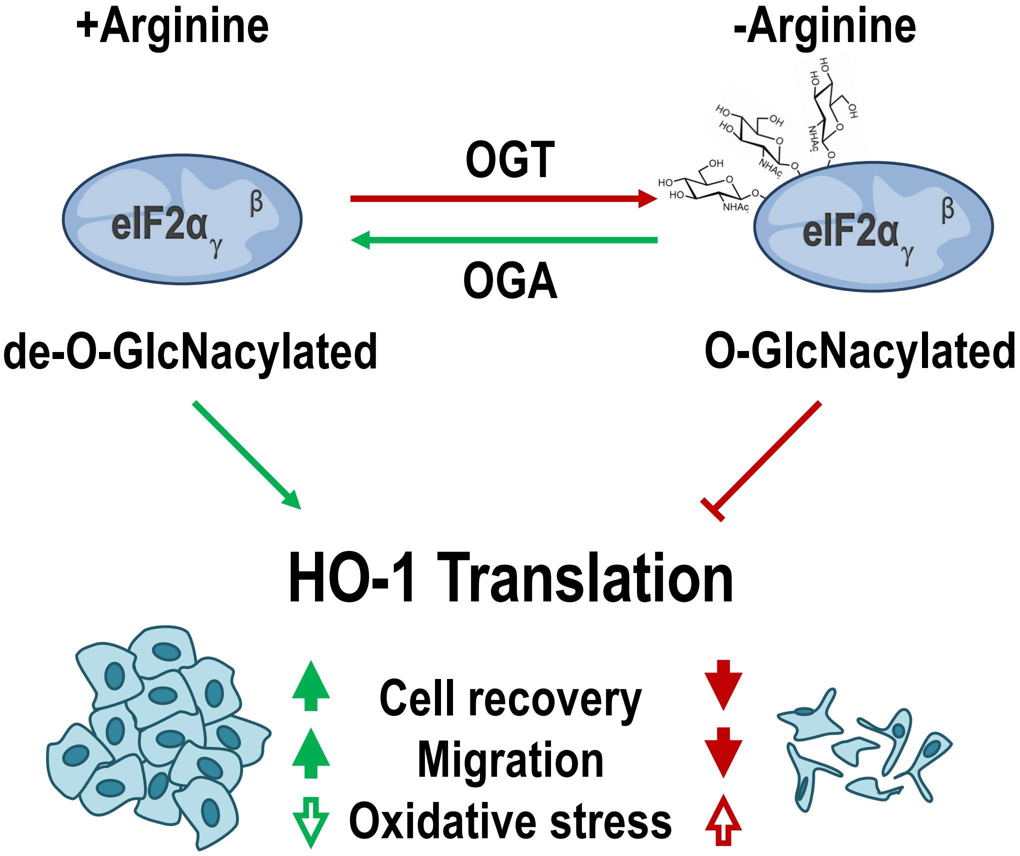

Supplemental Fig. S6

Fig. S6. Overall summary. Our data suggest that O-GlcNAcylation of eIF2α is the primary mechanism for downregulating HO-1 protein translation during ArgS. The downregulation of HO-1 protein leads to an increase in ROS levels and a reduction in cell recovery from ArgS and migratory ability. ArgS induces O-GlcNAcylation on eIF2α, which is considered a stress response to the treatment. The inhibitory effect of increased eIF2α O-GlcNAcylation on antioxidant protein translation reveals a novel mechanism by which ArgS may serve as a potential anti-cancer treatment.
