# Supplementary material for: Extracellular arginine availability modulates eIF2α O-GlcNAcylation and heme oxygenase 1 translation for cellular homeostasis
Source: J Biomed Sci. 2023 May 22;30:32. doi: 10.1186/s12929-023-00924-4 (PMC10201738; doi:10.1186/s12929-023-00924-4)
Supplement: Supplementary file 2 — Additional file 2: Table S1. Primer sequences for qRT-PCR. Table S2. Antibodies. Table S3. Primer sequences for mutagenesisof eIF2α. [file 12929_2023_924_MOESM2_ESM.pdf]

Table S1. Primer sequences for qRT-PCR

GAPDH	Forward: 5’- TGCACCACCAACTGCTTAGC-3'<br>Reverse: 5’- GGCATGGACTGTGGTCATGAG-3'
HMOX1	Forward: 5’- CCAGCAACAAAGTGCAAGATTC-3’<br>Reverse: 5’- TCACATGGCATAAAGCCCTACAG-3'
HK1	Forward: 5’-GTGAAAATCCGTAGTGGGAA-3'<br>Reverse: 5’-CTCTCTCCTTTTTATCGCAT-3'
HK2	Forward: 5’-CTTCTTCACGGAGCTCAACC-3'<br>Reverse: 5’-AAGCCCTTTCTCCATCTCCT-3'
GFPT	Forward: 5’-TGCTGTGCTGAGAGGCTATGATGT-3'<br>Reverse: 5’-GTCCAGAAATGCAACACCCAGCAT-3'
GPI	Forward: 5’-GATCCTCCTGGCCAACTTCT-3'<br>Reverse: 5’-GTTGGTTGGGCGATTTCCTT-3'
GNPNAT1	Forward: 5’-AGGACAGATTGTTGCTACGG-3'<br>Reverse: 5’-TTTGCCAAGCTGCTTTCCTC-3'
PGM3	Forward: 5’-ATCTTGGCTCTGAAGGGCTT-3'<br>Reverse: 5’-GCTTGTCTTTCAGCATCGGT-3'
NAGK	Forward: 5’-CAGTGTTGCCCGAGATTGAC-3'<br>Reverse: 5’-CAGGGTGAAGCTGGAGAAGA-3'
UAP1	Forward: 5’-TGTGGATGCACGAATGGAAC-3'<br>Reverse: 5’-TAGGATATGCAACGCCGAGT-3'

Table S2. Antibodies

ANTIBODIES	SOURCE	IDENTIFIER
anti-HO-1	Novus Biologicals	NBP1-97507
anti-eIF2α	Cell Signaling	#5324
anti-Phospho-eIF2α	Cell Signaling	#9721
anti-O-linked N-Acetylglucosamine (O-GlcNAc)	Abcam	ab2739
anti-O-GlcNAc transferase	Santa Cruz	sc-74547
anti-OGA/ NCOAT	Santa Cruz	sc-376429
anti-Histone H3	Active Motif	39763
anti-Actin	Millipore Sigma	MAB1501R
anti-Lamin A/C	Cell Signaling	#4777
anti-ATF4	Cell Signaling	#11815
anti-Biotin	Abcam	ab53494
anti-GSR	Proteintech	18257-1-AP
anti-CISD2	Proteintech	66082-1-Ig
anti-SOD1	Proteintech	67480-1-Ig
anti-DYKDDDDK tag (FLAG tag)	Proteintech	66008-3-Ig
anti-Mouse IgG, HRP-linked	Cell Signaling	#7076
anti-Rabbit IgG, HRP-linked	Jackson ImmunoResearch	211-032-171
Goat anti-Mouse IgG (H+L), Alexa Fluor 488	Thermo Fisher Scientific	A-11001
anti-Rabbit HQ	CRB DISCOVERY	760-4815
anti-HQ-HRP	CRB DISCOVERY	760-4820
anti-Mouse IgG1+IgG2a-IgG3	Abcam	ab133469

Table S3. Primer sequences for mutagenesis of eIF2α

S51A	Forward: 5’-CTTAGTGAATTAGCCAGAAGGCGTA-3'<br>Reverse: 5’-AAGAATCATGCCTTCAATGTT-3'
S219A	Forward: 5’-TTGAATTGTGCTACAGAAAACATGCCC-3'<br>Reverse: 5’-ACCTGCTCTTAGGGCTTCTTT-3'
T239A/<br>T241A	Forward: 5’-GTATGTAATGACTGCGACAGCCCTGGAGAGAACAGA<br>AG-3'<br>Reverse: 5’-CGAGGAGGAGCTATTAGATTAAT-3'
S219D	Forward: 5’-TTGAATTGTGATACAGAAAACATGCCC-3'<br>Reverse: 5’-ACCTGCTCTTAGGGCTTCTTT-3'
T239C/<br>T241C	Forward: 5’-GTATGTAATGACTTGTACATGCCTGGAGAGAACAGA<br>AG-3'<br>Reverse: 5’-CGAGGAGGAGCTATTAGATTAAT-3’
